# LKB1 is a central regulator of tumor initiation and pro-growth metabolism in ErbB2-mediated breast cancer

**DOI:** 10.1186/2049-3002-1-18

**Published:** 2013-08-14

**Authors:** Fanny Dupuy, Takla Griss, Julianna Blagih, Gäelle Bridon, Daina Avizonis, Chen Ling, Zhifeng Dong, Doris R Siwak, Matthew G Annis, Gordon B Mills, William J Muller, Peter M Siegel, Russell G Jones

**Affiliations:** 1 Goodman Cancer Research Centre, McGill University, Montréal, Québec, Canada; 2 Department of Biochemistry, McGill University, Montréal, Québec, Canada; 3 Department of Physiology, McGill University, McIntyre Building, Room 705, Montréal, Québec 3655, Canada; 4 Department of Medicine, McGill University, Room 513, 1160 Pine Avenue, West, Montréal, Québec, Canada; 5 Department of Systems Biology, The University of Texas MD Anderson Cancer Center, Houston, TX, USA

**Keywords:** Breast cancer, ErbB2, LKB1, Metabolism

## Abstract

**Background:**

Germline and somatic mutations in *STK11*, the gene encoding the serine/threonine kinase LKB1, are strongly associated with tumorigenesis. While loss of LKB1 expression has been linked to breast cancer, the mechanistic role of LKB1 in regulating breast cancer development, metastasis, and tumor metabolism has remained unclear.

**Methods:**

We have generated and analyzed transgenic mice expressing ErbB2 in the mammary epithelium of LKB1 wild-type or LKB1-deficient mice. We have also utilized ErbB2-expressing breast cancer cells in which LKB1 levels have been reduced using shRNA approaches. These transgenic and xenograft models were characterized for the effects of LKB1 loss on tumor initiation, growth, metastasis and tumor cell metabolism.

**Results:**

We demonstrate that loss of LKB1 promotes tumor initiation and induces a characteristic shift to aerobic glycolysis (‘Warburg effect’) in a model of ErbB2-mediated breast cancer. LKB1-deficient breast cancer cells display enhanced early tumor growth coupled with increased cell migratory and invasive properties *in vitro*. We show that ErbB2-positive tumors deficient for LKB1 display a pro-growth molecular and phenotypic signature characterized by elevated Akt/mTOR signaling, increased glycolytic metabolism, as well as increased bioenergetic markers both *in vitro* and *in vivo*. We also demonstrate that mTOR contributes to the metabolic reprogramming of LKB1-deficient breast cancer, and is required to drive glycolytic metabolism in these tumors; however, LKB1-deficient breast cancer cells display reduced metabolic flexibility and increased apoptosis in response to metabolic perturbations.

**Conclusions:**

Together, our data suggest that LKB1 functions as a tumor suppressor in breast cancer. Loss of LKB1 collaborates with activated ErbB2 signaling to drive breast tumorigenesis and pro-growth metabolism in the resulting tumors.

## Background

*STK11* was identified in 1998 as a novel tumor suppressor gene in patients with Peutz-Jeghers syndrome (PJS) [1], an autosomal, dominant disorder characterized by the presence of pigmented macules on the skin and mouth, coupled with the growth of benign polyps in the gastrointestinal tract [2]. While gastrointestinal tumors are the most common malignancies associated with PJS, patients also exhibit an 18-fold increased risk of developing epithelial cancers, including those of the breast [3]. The risk of developing breast cancer in PJS patients is 8% at the age of 40 and reaches 45% by the age of 70; which corresponds to a risk profile similar to patients with *BRCA1* and *BRCA2* mutations [3]. Consistent with these clinical observations, recent studies have linked LKB1 loss to enhanced breast cancer tumorigenesis in mice [4-6]. Indeed, the loss of LKB1, in the absence of a transforming oncogene, results in the emergence of mammary tumors with low penetrance and long latency [4]. Loss of LKB1 has been shown to accelerate mammary tumor formation in response to various oncogenes [5,6]. However, the functional role of LKB1 in restricting breast cancer initiation and growth is not fully understood.

The *STK11* gene encodes the protein kinase LKB1, a serine threonine kinase that plays a multi-faceted role in cell biology [7]. One of the best-characterized targets of LKB1 is the energy sensor AMP-activated protein kinase (AMPK). LKB1 phosphorylates and activates AMPK in response to energetic stress [8,9], leading to changes in cell metabolism designed to conserve cellular ATP. One of the main targets of LKB1 signaling is mTOR complex 1 (mTORC1). LKB1-dependent activation of AMPK inhibits mTORC1 activity via dual regulation of the tuberous sclerosis complex (TSC) [10] and the mTORC1 scaffold protein Raptor [11]. The diversity of LKB1-dependent biological functions may lie in the fact that LKB1 phosphorylates and regulates 12 AMPK-related kinases in addition to AMPK [12]. Given its diversity of kinase targets, LKB1 has been characterized as a ‘master’ kinase that regulates diverse cellular processes, including cell polarity, energy metabolism, apoptosis, and cell proliferation [7,13-15]. Importantly, all of these processes play a role in cancer initiation and progression, and may contribute at some level to the tumor suppressor effects of LKB1.

To investigate the functional role of LKB1 in breast cancer development and progression, we developed an LKB1-deficient mouse model of ErbB2-induced mammary tumorigenesis [16]. ErbB2 is a receptor tyrosine kinase overexpressed in 25% to 30% of human breast cancers, drives mammary tumor formation, and defines the HER2 subtype, a poor-prognosis form of breast cancer [17]. Here we report that deleting LKB1 expression in mammary epithelium harboring activating mutations in ErbB2 promotes increased tumor initiation and enhanced growth of early-stage mammary tumors. Reduced LKB1 expression is associated with diminished cell-to-cell contact and enhances the migratory and invasive properties of established ErbB2-driven breast cancer cells. Interestingly, LKB1-deficient ErbB2-positive tumors displayed a pro-growth molecular signature characterized by elevated Akt/mTORC1 and reduced AMPK signaling. LKB1-null, ErbB2-positive tumors displayed a metabolic phenotype characteristic of the Warburg effect *in vitro* and displayed heightened bioenergetic markers both *in vitro* and *in vivo.* Induction of the Warburg effect in these tumors is regulated, in part, by elevated mTORC1 signaling. Finally, the constitutive activation of mTORC1 signaling that accompanies LKB1 loss sensitizes breast cancer cells to apoptosis following metabolic challenge, such as glucose restriction. Together our data suggest that LKB1 loss cooperates with ErbB2 to promote primary tumor development and that loss of LKB1 signaling promotes a pro-growth metabolism of ErbB2-expressing breast cancer cells.

## Methods

### Transgenic mouse models

FVB mice bearing floxed *LKB1* alleles [18] were obtained from the National Cancer Institute (strain number: 01XN2). These mice were bred with MMTV/NIC transgenic mice previously generated in the laboratory of Dr. William J. Muller [19]. Mice were sacrificed when primary tumors reached maximal allowable volumes (6 to 8 weeks after the first palpation) and portions of each tumor were flash frozen in liquid nitrogen or fixed and embedded in paraffin. Mice were housed in facilities managed by the McGill University Animal Resources Centre and all animal experiments were conducted under ananimal use protocol approved by McGill University and developed in accordance with guidelines established by the Canadian Council on Animal Care.

### Cell lines, cell culture, and DNA constructs

The NIC cell line was established from a primary mammary tumor derived from the MMTV/NIC transgenic mouse [19]. Cells were maintained in DMEM supplemented with 5% FBS and 1× mammary epithelial growth supplement(Invitrogen, Burlington, ON, Canada). A shRNA targeting mouse LKB1 (sequence: 5′-AGGTCAAGATCCTCAAGAAGAA-3′) was cloned into the murine stem cell virus P2Gm plasmid (Addgene, 22699, Cambridge, MA, USA) using *Eco*RI and *Xho*I restriction sites. Retroviruses were generated in vesicular stomatitis virus cells according to the manufacturer’s instruction (CloneTech, Mountain View, CA, USA). Retrovirus-infected cells were selected by culture in 1 μg/ml puromycin and sorted by flow cytometry for green fluorescent protein (GFP) expression.

### 3D cell culture, migration and invasion assays

3D Matrigel cultures were established using 8-well chamber slides (NUNC, Rockford, IL, USA). In each well, 110 μl of Matrigel (BD Biosciences, Mississauga, ON, Canada) was plated and allowed to solidify at 37°C for 30 min. Subsequently, 1,000 mono-dispersed cells were mixed in 300 μl culture medium containing 10% FBS and 2% Matrigel and seeded on top of the Matrigel. The medium was changed every three days and cells analyzed after 9 days of culture by immunofluorescent staining.

Migration and invasion of NIC cells was assessed by plating 2 × 10^4^ and 1 × 10^5^ cells, respectively, in serum-free media, onto xCELLigence CIM plates (Roche Applied Science, Laval, QC, Canada). The rate of migration and invasion was monitored for 24 hours and calculated according to the manufacturer’s protocol. The data shown represent the average from three independent experiments performed in duplicate.

### *In vivo* tumor cell assays

To assess primary tumor growth, 1 × 10^6^ cells from each cell population (NIC-parental, NIC-FF (firefly luciferase)and NIC-LKB1 KD (knockdown)) were injected into the mammary fat pad (*n* = 10 animals per cell line) as previously described [20]. Experimental lung metastasis assays were conducted and the quantification of lung metastatic burden was performed as previously described [20]. Experimental metastasis assays were performed by injecting 5 × 10^5^cells directly into the lateral tail vein of severe combined immunodeficiency(SCID)/beige mice (*n* = 10 animals per cell line). All mice were sacrificed four weeks post-injection. The lung metastatic burden was also determined.

### Reverse phase protein arrays (RPPA)

Tumor lysates were prepared and processed following the protocol available on the MD Anderson Cancer Center website [21]. Statistical analysis of the RPPA data is described in Additional file 1.

### Immunoblotting

NIC breast cancer cells were cultured to 80% confluency and lysed in ice cold AMPK lysis buffer [22] supplemented with protease and phosphatase inhibitors (Roche, Laval, QC, Canada), dithiothreitol(1 μg/ml), and benzamidine (1 μg/ml). Immunoblots were performed as previously described [20] using several primary antibodies (Additional file 2: Table S1).

### Analysis of metabolites by liquid chromatography and mass spectrometry (LC-MS)

Tumor samples (50 mg), or cell lines cultured for 24 hours, were extracted in a solution of 50% acetonitrile (ACN) and injected onto an Agilent 6430 triple quadrupole LC-MS system for targeted metabolite analysis (ATP, ADP, AMP, creatine, glucose, and lactate). Chromatography was performed using a 1290 Infinity ultra-performance liquid chromatography system (Agilent Technologies, Santa Clara, CA, USA) consisting of vacuum degasser, auto-sampler and a binary pump. The column temperature was maintained at 10°C and the injection volume was 5 μl. Separation was achieved using a Cogent Diamond Hydride column (4.0 μm, 2.1 × 100.0 mm) (MicroSolv Technology, Eatontown, NJ, USA) using a flow rate of 0.4 ml/min and a Cogent Diamond Hydride guard column (4.0 μm, 2.0 × 20.0 mm) (MicroSolv Technology, Eatontown, NJ, USA). The chromatography run started with a 2 minute hold in 97% solution B (15 mM ammonium formate in 85% ACN/15% H_2_O, pH 5.8) and 3% solution A (15 mM ammonium formate in H_2_O, pH 5.8). Subsequently, samples were subjected to a 5 minute gradient down to 70% solution B, followed by a 3 min step with 98% solution A. A subsequent re-equilibration time (6 min) was performed prior to the next injection. The mass spectrometer is equipped with an electrospray ionization source and samples were analyzed in positive mode for creatine, glucose, and lactate and in negative mode for ATP, ADP, and AMP. Data were quantified by integrating the area under the curve of each optimized multiple reaction monitoring transition using authentic standards for each metabolite. Absolute quantification was performed using calibration curves processed with Agilent MassHunter Quantitative Analysis software. Transitions in negative ionization mode for quantifier and qualifier ions were, respectively: 506.0 → 158.9 and 506.0 → 78.9 for ATP; 426.0 → 134 and 426.0 → 79 for ADP; 346.0 → 97 and 346.0 → 78.9 for AMP; 179.0 → 89.0 and 179 → 59 for glucose; and 89 → 43.1 for lactate. Transitions in positive ionization mode were 132.0 → 44.2 and 132.0 → 90.1 for creatine. The gas temperature was 350°C, the flow rate was 10 l/min, the nebulizer pressure was 50 psi (≈0.34 MPa),and the capillary voltage was +4000 V. Total amounts of each metabolite were normalized per mg of tumor tissue or per cell number as indicated.

### Respirometry

The oxygen consumption rate (OCR) and extracellular acidification rate (ECAR) of cells were measured using an XF24 Extracellular Flux Analyzer (Seahorse Bioscience, Massachusetts, USA). In brief, cells were plated at 5 × 10^*5*^/well, starved overnight and stimulated with 5% serum for 6 hours. Rates were measured as previously described [23] at baseline levels. The data shown correspond to one representative experiment out of three performed and the values represent an average of six wells.

### Analysis of metabolites by gas chromatography and mass spectrometry (GC-MS)

Water-soluble metabolites were extracted (5 × 10^6^cells/10 cm dish) and prepared for analysis as described previously [24,25]. Tricarballylic acid was added as an internal standard following cell lysis. Extracts were dried using a chilled vacuum centrifuge (Labconco, Kansas City, MO USA) and stored at −80°C until GC-MS analysis. Samples were resuspended in 30 μl anhydrous pyridine and 70 μl *N*-*tert*-butyldimethylsilyl-*N*-methyltrifluoroacetamide and incubated at 70°C for 1 hour. The GC-MS data were acquired on an Agilent 5975C series GC/MSD system (Agilent Technologies, Santa Clara, CA, USA) operating in electron ionization mode (70 eV) for selected ion monitoring. The relative amount of each metabolite was determined from the integral ratios of the metabolites to the internal standard and normalized to the number of cells extracted. The amount of each reported metabolite was normalized to the number of cells (nM/10^6^ cells).

### Measurement of glucose uptake

Glucose uptake was determined using the fluorescent glucose analog 2-(N-(7-nitrobenz-2-oxa-1,3-diazol-4-yl)amino)-2-deoxyglucose (2-NBDG) following the manufacturer’s instructions (Invitrogen, Burlington, ON, Canada). Briefly, cells were incubated with 100 nM 2-NBDG for 45 minutes and the mean fluorescent intensity was measured in the FL-2 channel using a Gallios flow cytometer (Beckman Coulter). The mean fluorescent intensity was normalized to the basal cellular GFP fluorescence to correct for differences in GFP expression between cell lines. The values represent an average of triplicate samples for each experiment.

### RNA extraction, cDNA synthesis, and quantitative PCR

RNA extraction, cDNA synthesis, and quantitative PCR were performed as previously described [26]. The list of primers is provided in Additional file 3: Table S2. Experiments were performed in triplicate using three different cDNA preparations. In each experiment, the NIC-FF sample served as the reference sample and Rpl13 was used as the control. Real-time PCR was performed using an Applied Biosystems 7500 instrument (Applied Biosystems, Burlington, ON, Canada). The data are represented as the relative mRNA expression in LKB1 knockdown cells compared with control cells for each individual gene.

### Measurement of lactate production

Lactate in culture medium collected from cells cultured for 48 hours, with or without rapamycin treatment (100 nM), was determined using either the NOVA BioProfile 400 analyzer or the Eton Bioscience kit (Eton Bioscience, Charlestown, MA,USA) according to the manufacturer’s instructions. In each case, the resulting data were normalized to the cell number.

### Cleaved caspase-3 assay

To assess tumor cell apoptosis in response to metabolic challenge, 6,000 cells were plated in a 96-well plate and maintained for 24 hours prior to switching to experimental culture conditions, which included 25 mM or 1 mM glucose each in the presence or absence of rapamycin (100 nM) for 72 hours. Cells were subsequently fixed in 4% paraformaldehyde and washed in PBS with 0.1% Triton. Endogenous peroxidases were quenched by treatment with wash buffer plus 1% H_2_O_2_. A cleaved caspase-3 antibody (Cell Signaling Technology, #9661, Whitby, ON, Canada) was applied for 1 hour at room temperature (1:250), followed by a 1 hour incubation with secondary HRP-conjugated, anti-rabbit antibodies. Chemiluminescent reagent was then added and detected using a plate reader. The resulting chemiluminescent signals were then normalized to cell number, which was determined by performing a crystal violet staining of the plate and reading the absorbance at 595 nm. The data shown correspond to one representative experiment out of three performed and the values represent an average of six wells.

### Viability assay

To assess tumor cell apoptosis in response to metabolic challenge, cells were plated in 12-well plates and maintained for 24 hours. Cultures were then switched to the experimental conditions, which included 25 mM or 1 mM glucose each in the presence or absence of rapamycin (100 nM) for 72 hours. Cells were then incubated with 7-AAD (eBioscience, San Diego, CA, USA) at 5 μl per sample for 15 min and the fluorescence was detected with a Gallios flow cytometer in the FL-4 channel (Beckman Coulter). The percentage of dead cells (7-AAD positive) was calculated using FlowJo software (Tree Star Inc.). The data represent one experiment out of three independent replicates, and are the mean ± standard deviation for triplicate samples.

### Statistical analysis

Statistical analyses were run using a two-tailed Student’s *t*test and online software (VassarStats) and the *P *values were represented using the following annotation: *, *P*< 0.05; **, *P*< 0.01; ***, *P*< 0.001. The significance of the tumor growth curves was assessed using analysis of variance and significance was indicated when the knockdown population was statistically different from both the parental and the control cell lines. Data are expressed as mean ± standard deviation for *n* ≥ 3.

## Results

### Loss of LKB1 enhances the development of ErbB-2-induced mammary tumors

**Figure 1:**
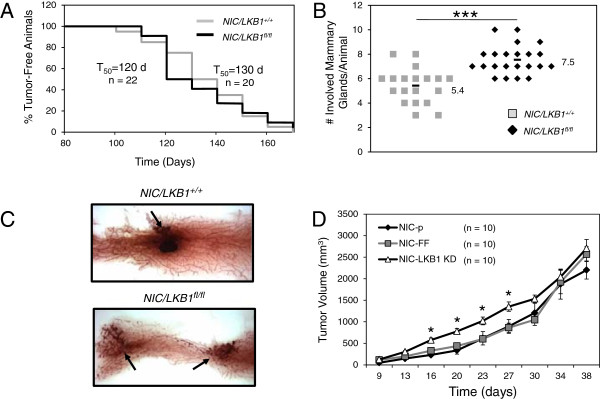
**LKB1 loss promotes the initiation of ErbB2-induced mammary tumors. (A)** Kaplan-Meier analysis, depicting the percentage of tumor-free animals over time in NIC/LKB1^+/+^and NIC/LKB1^fl/fl^ cohorts. The *T*_50_ values represent the time at which 50% of the mice develop their first palpable mammary tumor. *n*, number of animals analyzed in each cohort. **(B)** Number of tumor-bearing mammary glands in each cohort. The average number of involved glands is increased in NIC/LKB1^fl/fl^ (7.5 ±1.1) compared with NIC/LKB1^+/+^ mice (5.4 ±1.4) (***, *P*< 0.001). **(C)** Hematoxylin staining of mammary gland whole mounts dissected from 3-month-old NIC/LKB1^+/+^ and NIC/LKB1^fl/fl^ mice. Arrows indicate the presence of pre-neoplastic lesions. **(D)** Mammary tumor growth following mammary fat pad injection of NIC tumor cells harboring shRNAs targeting FireFly luciferase (NIC-FF) and NIC mammary tumors with stable LKB1 knockdown (NIC-LKB1 KD). NIC-FF or NIC-LKB1 KD cells were injected in the mammary fat pads of 8-week old SCID/beige mice (*n* = 10 mice per group) and tumor growth was monitored by bi-weekly calliper measurements (*, *P*< 0.05).

Deletion of LKB1 in the mouse mammary gland has been shown to result in spontaneous tumor formation with low penetrance (19%) and long latency (46 to 85 weeks) [4]. To examine the consequences of LKB1 loss on mammary tumor formation driven specifically by the ErbB2 oncogene, we crossed mice containing a floxed LKB1 gene [18] with a MMTV/NIC mouse model that expresses an activated form of ErbB2 (NDL2-5) and Cre recombinase from a single bicistronic transgene [19]. This genetic cross was performed to ensure that deletion of LKB1 occurred in every mammary tumor cell transformed by ErbB2 *in vivo*. No significant difference in tumor onset was observed in NIC/LKB1^+/+^ mice (*T*_50_ = 130 days) when compared with NIC/LKB1^fl/fl^ animals (*T*_50_ = 120 days), with both cohorts developing palpable tumors at 17 to 18 weeks of age (Figure 1A). However, we did observe that the number of tumor-bearing mammary glands was significantly higher in LKB1 mutant mice, with an average of 7.5 involved glands in NIC/LKB1^fl/fl^ mice compared with 5.4 involved glands for NIC/LKB1^+/+^ animals (Figure 1B). This result suggests that loss of LKB1 promotes an increase in the number of ErbB2-dependent transformation events leading to increased overall tumor formation.

To determine whether the loss of LKB1 is associated with enhanced tumor initiation, we examined inguinal mammary gland whole mounts of 3-month-old mice (prior to the average age at first tumor palpation). This analysis revealed an increase in the number of pre-neoplastic lesions in mammary glands of NIC/LKB1^fl/fl^ mice compared with NIC/LKB1^+/+^animals (Figure 1C). To further examine the effects of LKB1 loss on mammary tumor growth, we used a mammary tumor cell explant model (herein called ‘NIC’) derived from an MMTV/NIC transgenic mouse [19]. The NIC cell line was infected with retroviruses containing control shRNAs (targeting firefly luciferase, FF) or shRNAs against LKB1 (LKB1 KD). Immunoblot analyses revealed that LKB1 levels were effectively diminished in explanted LKB1 shRNA-expressing NIC mammary tumor cells compared with NIC cells expressing the control shRNA (see Additional file 4: Figure S1). We next examined the level of suppression of LKB1-dependent signaling in our shRNA-expressing NIC cell lines by assessing their responses to metabolic stressors known to activate LKB1-dependent pathways. Metformin is an inhibitor of complex I in the mitochondria [27,28], and leads to LKB1-dependent activation of AMPK [9]. Following metformin treatment, both parental and control NIC cells displayed an increase in AMPK phosphorylation at T172 and elevated phosphorylation of the AMPK target acetyl-CoA carboxylase (ACC) (Additional file 4: Figure S1). By contrast, LKB1 shRNA-expressing NIC cells displayed reduced levels of both p-AMPK and pACC relative to control cells, indicating reduced signaling downstream of LKB1.

To determine the effect of diminished LKB1 levels on mammary tumor growth *in vivo*, we injected parental (NIC-p), control (NIC-FF). and LKB1 KD (NIC-LKB1 KD) cells into the mammary fat pads of immunocompromised mice. NIC cells expressing LKB1 shRNA displayed accelerated growth at early time points post-injection compared with NIC-p and NIC-FF tumor cells (Figure 1D). However, this initial difference in the growth of LKB1-deficient tumors was progressively lost after 30 days (Figure 1D).

### Reduced LKB1 expression in breast cancer cells promotes an invasive phenotype

**Figure 2:**
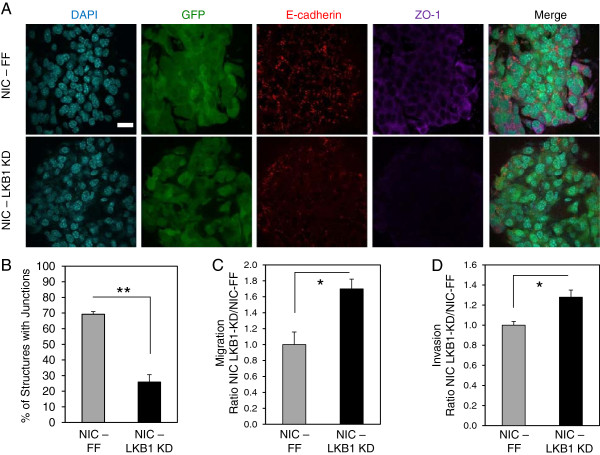
**LKB1 knockdown in breast cancer cells causes reduced expression of epithelial markers and acquisition of migratory and invasive properties. (A)** Representative immunofluorescent images of NIC-FF and NIC-LKB1 KD cells in 3D collagen cultures stained with E-cadherin (red) and ZO-1 (purple). Nuclei were counterstained with 4',6-diamidino-2-phenylindole (DAPI) (blue) and GFP images are shown to confirm that breast cancer cells retain expression of the control or LKB1-targeting shRNAs. The scale bar in the upper left inset represents 20 μm and applies to all panels. **(B)** Quantification of the number of cell colonies exhibiting strong junctional protein expression (E-cadherin and ZO-1 staining). The data correspond to an average of three independent experiments. **, *P*< 0.01. The migratory **(C)** and invasive **(D)** rates of NIC-FF and NIC-LKB1 KD cells were assessed using the xCELLigence platform. The data represent an average of three independent experiments performed in duplicate. *, *P*< 0.05.

To characterize the effect of LKB1 loss on tumor cell phenotype further, we next investigated the status of cell polarity in ErbB2-positive breast cancer cells with reduced LKB1 expression. Loss of cell polarity is an important characteristic for the acquisition of migratory and invasive phenotypes [29,30] and LKB1 has been previously linked with the regulation of polarity in a variety of cellular contexts [31,32]. Mice deficient for LKB1 do not survive beyond embryonic day E10.5, owing to impaired production of vascular endothelial growth factor and defective vascular development[33]. To investigate the biological impact of LKB1 down-regulation on junction formation and cellular adhesion *in vitro*, we analyzed 3D Matrigel cultures of control NIC cells and NIC cells expressing LKB1 shRNA using confocal microscopy. By contrast with control NIC cells, which grow cohesively and exhibit high levels of E-cadherin and ZO-1 expression, mammary tumor cells with reduced LKB1 expression displayed fewer cell contacts and expressed significantly lower levels of both junctional markers (Figure 2A). Our analysis of cell colonies that exhibited strong E-cadherin and ZO-1 staining revealed that NIC cells with reduced LKB1 expression produced 60% fewer acini with substantial junctional protein expression (E-cadherin and ZO-1) (Figure 2B), indicating that reduced LKB1 levels correlate with reduced cell junction integrity. To assess whether loss of LKB1 affects the migration and invasion properties of ErbB2-dependent tumor cells, we analyzed the behavior of NIC-FF and NIC-LKB1 KD cells *in vitro* using the xCELLigence platform. LKB1 shRNA-expressing NIC cells exhibited enhanced migration (Figure 2C) and invasion (Figure 2D) rates compared with control tumor cells; this is evidence of a higher invasive phenotype, and is consistent with the diminished junctional integrity exhibited by these cells.

### Loss of LKB1 results in reduced lung metastatic burden

**Figure 3:**
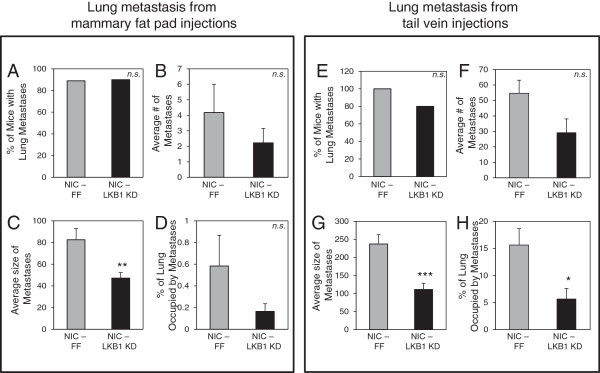
**Loss of LKB1 results in reduced lung metastatic burden.** The percentage of mice within each cohort that developed spontaneous lung metastases, the number of lung metastases, the average lesion size and the percentage lesion area present per total lung area were determined at necropsy on mice subjected to mammary fat pad injection with NIC-FF and NIC LKB1 KD cells (*n* = 10 mice per group) **(A-D)** or mice subjected to tail vein injections with NIC-FF and NIC LKB1 KD cells (*n* = 10 mice per group) **(E-H)**. *, *P*< 0.05; **, *P*< 0.01; ***, *P<* 0.001. *n.s.*, not significant.

Reduced cell-to-cell contacts and increased migration and invasion are critical features for efficient metastasis [29,30]. To assess the impact of LKB1 loss on breast cancer metastasis, we examined the lung metastatic burden of mice previously subjected to mammary fat pad injections with NIC-FF or NIC-LKB1 KD cells (*n* = 10 animals per cell line). Approximately 90% of animals for both cohorts developed lung metastases (Figure 3A). However, amongst animals presenting lung metastases, the number of metastases per lung, though lower, was not significantly affected by the reduction of LKB1 (Figure 3B). Interestingly, we observed that the size of individual metastases present in the lung of animals injected with NIC cells expressing LKB1 shRNA was significantly diminished (Figure 3C), which resulted in a significantly reduced percentage of lung surface covered by lesions (Figure 3D).

To investigate this issue further, we conducted an experimental metastasis assay by injecting NIC tumor cells expressing control (FF) or LKB1 shRNA (LKB1 KD) via the tail vein (*n* = 10 animals per cell line), and assessed subsequent seeding of tumor cells in the lung. All mice injected with control NIC breast cancer cells developed lung metastases, compared with 80% of animals injected with NIC-LKB1 KD cells developing lung metastases (Figure 3E). Like the spontaneous metastasis assay, the number of lung metastases per lung was not significantly affected by the status of LKB1 (Figure 3F). However, both the average size of individual metastases (Figure 3G) and the percentage of lung tissue occupied by metastatic lesions (Figure 3H) were substantially reduced in animals that received LKB1 shRNA-expressing tumor cells, compared with mice that received control NIC tumor cells.

### LKB1-deficient mammary tumors display a pro-growth molecular signature

**Figure 4:**
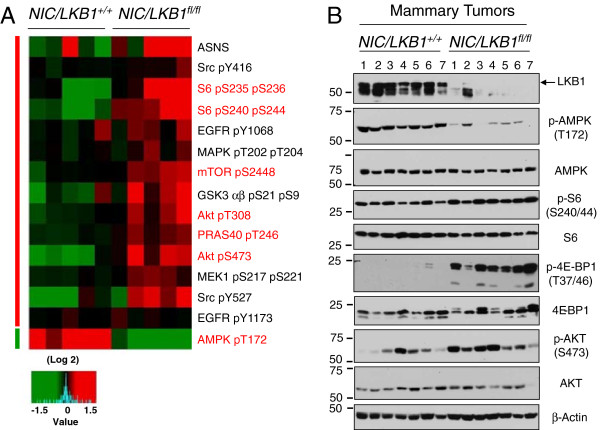
**LKB1 loss confers a pro-growth signal transduction signature in ErbB2-positive mammary tumors. (A)** Five NIC/LKB1^+/+^and five NIC/LKB1^fl/fl^ mammary tumors were subjected to RPPAanalysis. Expression of selected proteins and phospho-proteins that are differentially expressed between NIC/LKB1^+/+^ and NIC/LKB1^fl/fl^ mammary tumors. Color key indicates level of expression, with green signifying proteins and phospho-proteins that are underexpressed and red identifying those that are overexpressed compared with control cells. **(B)** Immunoblot analysis of mammary tumor lysates derived from NIC/LKB1^+/+^and NIC/LKB1^fl/fl^ mice with antibodies directed to components of the mTOR and Akt signaling pathways. Immunoblot analysis for β-actin serves as a loading control.

To investigate molecular mechanisms contributing to the pro-growth, invasive phenotype of LKB1-deficient ErbB2-positive breast cancer cells, we examined primary tumors using a reverse phase protein array (RPPA). Five tumors from each genotype (NIC/LKB1^+/+^ and NIC/LKB1^fl/fl^) were analyzed using a panel of 126 antibodies [34] (Additional file 5: Table S3). Unsupervised clustering of the data resulted in a clear segregation between mammary tumors from NIC/LKB1^+/+^ mice and those arising in NIC/LKB1^fl/fl^ animals (see Additional file 6: Figure S2 and Additional file 1: description of the statistical analysis). As expected, one of the largest differences was seen in the phosphorylation status of AMPK, which was severely reduced in all NIC/LKB1^fl/fl^ tumors (Figure 4A). This result is consistent with the role of LKB1 as an upstream activator of AMPK [8,9].

LKB1 has been shown to mediate some of its effects on tumorigenesis through modulation of the mTOR pathway [32,35]. We observed an increase in ribosomal S6 protein phosphorylation at both S235/236 and S240/244, suggesting increased mTOR activity in LKB1-deficient ErbB2-positive tumors (Figure 4A). Interestingly, LKB1-deficient tumors also displayed increased Akt activation, as shown by elevated phosphorylation levels of T308 and S473 in Akt (Figure 4A). Evidence of elevated mTOR phosphorylation on S2448 and elevated GSK3 phosphorylation at both S9 and S21, which are all known Akt phosphorylation sites, was also detected in NIC/LKB1^fl/fl^ tumors (Figure 4A).

We next validated the results from the RPPA analysis by immunoblotting mammary tumor lysates derived from NIC/LKB1^+/+^ and NIC/LKB1^fl/fl^ mice. As expected, LKB1 was readily detected in mammary tumors harvested from NIC/LKB1^+/+^ mice but absent in NIC/LKB1^fl/fl^ mammary tumors (Figure 4B). In agreement with our RPPA analysis (Figure 4A), reduced levels of phosphorylated AMPK were detected in LKB1-deficient tumors (Figure 4B). These phenotypes were also confirmed by immunohistochemistry (*n* = 4 mammary tumors from each cohort) (see Additional file 7: Figure S3). Activation of Akt in LKB1-deficient mammary tumors was also confirmed using phospho-Akt antibodies (Figure 4B). LKB1-deficient mammary tumors displayed elevated levels of mTORC1 pathway activation, as measured by increased pS6 and hyperphosphorylated 4E-BP1 by immunoblot analysis (Figure 4B) and immunohistochemistry (see Additional file 8: Figure S4 and Additional file 1: description of methods). Interestingly, immunohistochemistry revealed that loss of LKB1 did not increase the intensity of staining for pS6 and p4E-BP1 in mammary tumors; rather the total number of cells exhibiting positive staining for mTORC1 activity markers was elevated in mammary tumors lacking LKB1 (see Additional file 8: Figure S4). Collectively these results indicate that tumors lacking LKB1 display a protein activation signature associated with pro-growth PI3K/Akt/mTOR signaling.

### Loss of LKB1 promotes the Warburg effect and increased bioenergetics in ErbB2-positive mammary tumors

**Figure 5:**
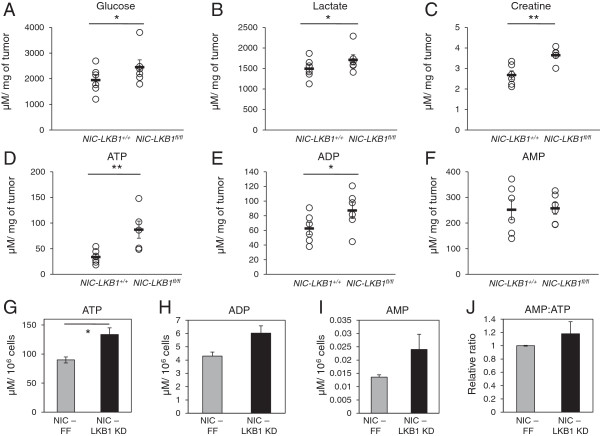
**Loss of LKB1 results in increased bioenergetic markers in ErbB2-positive mammary tumors and derived cell lines. (A-F)** Six NIC/LKB^+/+^ and six NIC/LKB1^fl/fl^ mammary tumors were subjected to LC-MS analysis. Intracellular levels of glucose **(A)**, lactate **(B)**, creatine **(C)**, ATP **(D)**, ADP **(E)**, and AMP **(F)** are represented as μM per mg of tumor. **(G-J)** NIC-FF and LKB1 KD cells were subjected to metabolic analyses as in **(A-F)**. Intracellular levels of ATP **(G)**, ADP **(H)** and AMP **(I)** are represented in μM per 10^6^ cells. The AMP:ATP ratio **(J)** in LKB1 KD cells is expressed relative to the ratio for control cells. *, *P*< 0.05; **, *P*< 0.01.

Our results from Figure 4 suggest that mammary tumors lacking LKB1 display increased mTOR and Akt signaling. Besides its well-characterized role in promoting cell survival and growth, Akt/mTOR signaling can also stimulate metabolic pathways, such as aerobic glycolysis in cancer cells [36]. To assess the effects of LKB1 loss on the metabolism of ErbB2-positive tumors, we characterized the bioenergetic profiles of primary ErbB2-positive tumors using LC-MS analysis (Figure 5). LKB1-deficient breast tumors displayed increased intracellular levels of glucose (Figure 5A) and lactate (Figure 5B), both hallmarks of the Warburg effect [37]. We also observed statistically higher levels of energy storage molecules, including creatine (Figure 5C), ATP (Figure 5D), and ADP (Figure 5E) in LKB1-deficient tumors, while the levels of AMP, which is a low-energy metabolite, were not affected by LKB1 status (Figure 5F). We next confirmed these results in our cell line models. We observed a 50% increase in the level of ATP (Figure 5G) in LKB1-deficient cells compared with control cells; the levels of ADP (Figure 5H) and AMP (Figure 5I), although higher in LKB1 KD cells, were not statistically significant. Consequently, the AMP: ATP ratio was not statistically different in cells with normal or reduced LKB1 expression (Figure 5J). Together these data indicate that LKB1 loss enhances the bioenergetic profile of primary mammary tumors.

**Figure 6:**
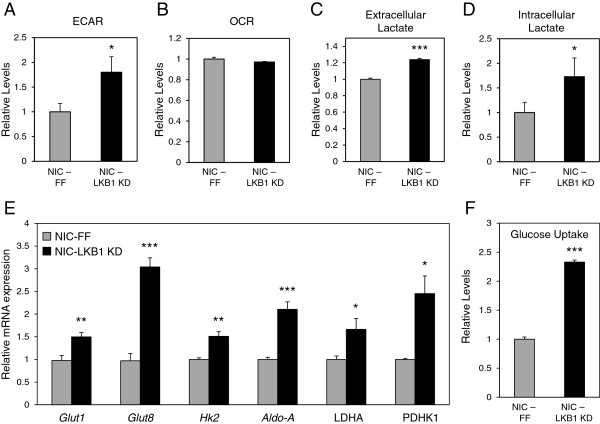
**LKB1-deficient breast cancer cells display increased aerobic glycolysis. (A-B)** Extracellular flux analysis of NIC cell lines. NIC-FF and NIC-LKB1 KD cells were plated for ECAR (A) and OCR (B) analyses. The data represent one representative experiment of three independent replicates and the values correspond to an average of five wells per experiment. **(C-D)** NIC-FF and NIC-LKB1 KD cells were cultured for 48 hours and the relative extracellular (C) and intracellular (D) levels of lactate were determined using an enzymatic assay and GC-MS, respectively. The data represent one representative experiment from three independent replicates, each performed in triplicate. **(E)** Total RNA was isolated from NIC-FF and NIC-LKB1 KD cells, and the relative mRNA expression of several glycolytic enzymes was determined by qPCR. Transcript levels were determined relative to *Rpl13* (60S ribosomal protein L13) mRNA levels, and normalized relative to its expression in control NIC-FF cells. The data represent the average of three independent experiments, each performed in triplicate. **(F)** Glucose uptake in NIC-FF and NIC-LKB1 KD cells was measured by flow cytometry using the 2NBDG fluorescent glucose analog. The data correspond to one representative experiment out of four independent replicates, each performed in triplicate. *, *P*< 0.05; **, *P*< 0.01; ***, *P*< 0.001.

To assess the impact of LKB1 loss on the metabolism of ErbB2-positive breast tumors, we conducted bioenergetic profiling of NIC tumor cells *in vitro* using an extracellular flux analyzer. NIC cells with reduced LKB1 expression (NIC-LKB1 KD) displayed a two-fold increase in their elevated ECAR, an index of lactate production [38], relative to control NIC cells (ECAR, Figure 6A). However, loss of LKB1 did not affect oxygen consumption by NIC cells (OCR, Figure 6B), consistent with observations that oxygen consumption is largely normal in cells undergoing the Warburg effect [39]. Consistent with an increased ECAR, LKB1-deficient NIC cells displayed an increase in both extracellular (Figure 6C) and intracellular (Figure 6D) lactate levels when compared with control tumor cells.

We next examined the expression of genes encoding enzymes involved in glycolysis using quantitative PCR. LKB1-deficient cells displayed a significant increase in the expression of several genes associated with glycolysis including *Glucose transporter 1* (*Glut1*), *Glucose transporter 8* (*Glut8*), *Hexokinase 2* (*Hk2*), *Aldolase A* (*Aldo-A*), *Lactate dehydrogenase A* (*LDHA*) and *Pyruvate dehydrogenase kinase 1* (*PDHK1*) (Figure 6D). The elevated levels of lactate and increased expression of glycolytic genes observed in LKB1-depleted NIC cells is consistent with the increased glucose and lactate levels observed in primary mammary tumors (Figure 5). We then assessed the significance of increased glucose transporter expression by measuring the level of glucose uptake by our NIC cells. Using the 2NBDG fluorescent glucose analog, we demonstrate that LKB1-deficient cells exhibit a 2.3-fold increase in glucose uptake relative to control NIC cells. This result is in agreement with the observed increase in lactate production when LKB1 expression is reduced.

### Loss of LKB1 sensitizes breast cancer cells to metabolic stress

**Figure 7:**
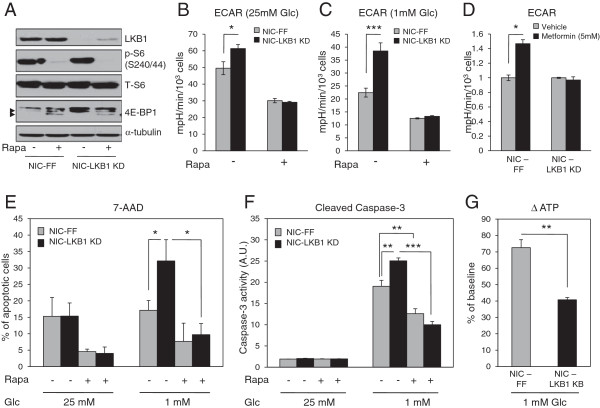
**Loss of LKB1 sensitizes cells to metabolic stress. (A)** Immunoblotting was performed on protein extracts from NIC-FF and NIC-LKB1 KD cells treated with rapamycin (100 nM) for 24 hours to confirm mTOR activity. Arrows point to the hypophosphorylated forms of 4E-BP1. **(B-C)** ECAR analysis of NIC-FF and NIC-LKB1 KD cells treated or not with rapamycin for 24 hours in full glucose conditions **(B)** or in 1 mM glucose conditions **(C)**. **(D)** ECAR analysis of NIC-FF and NIC-LKB1 cells treated with vehicle or with metformin (5 mM) for 6 hours. **(E-F)**. Viability assays were performed on NIC-FF and NIC-LKB1 KD cells cultured in 25 mM glucose or 1 mM glucose and treated or not treated with rapamycin for 72 hours. The level of apoptosis was assessed using 7-AAD **(E)** or cleaved caspase 3 levels **(F)**. All graphs correspond to a representative experiment of three performed, and values represent the average of six wells for **(B)**, **(C)**, **(D)** and **(F)** and three wells for **(E)**. **(G)** NIC-FF and NIC-LKB1 KD cells were cultured for 24 hours in 1 mM glucose and intracellular ATP levels were quantified as the percentage drop from baseline conditions (25 mM of glucose). *, *P*< 0.05; **, *P*< 0.01; ***, *P*< 0.001.

Cells with deregulated mTOR signaling, such as TSC2-null cells, gain a metabolic growth advantage, but also display increased sensitivity to metabolic stresses including glucose deprivation [40]. ErbB2-driven breast tumors lacking LKB1 display elevated mTORC1 activity (Figure 4). We first validated that the selected concentration of rapamycin and duration of treatment were sufficient to inhibit mTOR activity in our cells. Immunoblot analysis confirmed the efficacy of the inhibitor, as revealed by the loss of S6 phosphorylation and the accumulation of hypophosphorylated forms of 4E-BP1 (Figure 7A). To assess whether the loss of LKB1 leads to mTOR-dependent glucose addiction in breast cancer, we analyzed the glycolytic profile of NIC tumor cells in response to glucose availability. Under full glucose conditions, NIC cells expressing LKB1 shRNA displayed an elevated ECAR compared with control cells (Figure 7B). Reducing glucose in the culture medium to 1 mM lead to a 60% drop in the ECAR of control cells (Figure 7C); importantly, LKB1-deficient cells dropped their ECAR by only 40% in response to low glucose and maintained an ECAR roughly equivalent to control cells under full growth conditions (Figure 7B, C). The enhanced lactate production by LKB1-deficient NIC cells was dependent on mTORC1 activity, as the ECAR of control and LKB1 KD cells was equivalent when cells were treated with the mTORC1 inhibitor rapamycin (Figure 7B, C). Direct measurement of extracellular lactate levels confirmed an increase in lactate production by LKB1-deficient NIC cells and the ablation of this glycolytic phenotype by rapamycin treatment (see Additional file 9: Figure S5). These data suggest that mTORC1 signaling contributes to the glycolytic phenotype observed in LKB1-deficient ErbB2-positive tumor cells.

To study the metabolic flexibility of breast cancer cells lacking LKB1, we treated ErbB2-positive tumor cells with the complex I inhibitor metformin and investigated its impact on ECAR. Immunoblot analysis confirmed that metformin increased AMPK phosphorylation over time, which was coincident with reduced S6 phosphorylation and accumulation of the hypophosphorylated form of 4E-BP1 (see Additional file 10: Figure S6). Metformin could suppress mTORC1 activity in both control and LKB1 KD cells, although this effect was delayed in cells expressing LKB1 shRNA. While metformin stimulated an increase in glycolysis in control tumor cells, LKB1-deficient cells were unable to respond to metformin by increasing their ECAR (Figure 7D).

Finally, we assessed the apoptotic response of LKB1-deficient NIC cells in response to energetic stress. Tumor cells lacking LKB1 displayed increased apoptosis, as measured by 7-AAD (Figure 7E) and Caspase-3 activation (Figure 7F), when cultured under low glucose conditions. Rapamycin protected LKB1-deficient NIC cells from apoptosis induced by glucose deprivation (Figure 7E and 7F), suggesting that dampening mTORC1 signaling in these tumor cells can confer protection from nutrient limitation. In agreement with an increased sensitivity to glucose depletion, we observed a more dramatic drop in intracellular ATP levels within LKB1-deficient cells (72% reduction) compared with the control cells (40% reduction) when cultured for 24 hours in 1 mM glucose (Figure 7G). These results suggest that LKB1-deficient cells are unable to maintain cellular ATP levels in response to energetic stress, promoting an increase in cell death.

## Discussion

LKB1 is a central growth-regulatory kinase that exerts its effects, in part, through the negative regulation of pro-growth pathways such as mTOR. LKB1 is a well-established tumor suppressor, with both germline and somatic mutations in *STK11*, the gene encoding LKB1, associated with cancer development. While broadly linked with cancer, LKB1’s role in breast cancer development and metabolic regulation in primary tumors has been poorly understood. To address this question, we created a genetically engineered mouse model to assess the impact of LKB1 deletion on the development and progression of breast tumors driven by the ErbB2 oncogene. We observed that ErbB2-mediated breast tumorigenesis is enhanced by LKB1 deletion, which is consistent with both experimental and clinical data linking LKB1 to breast cancer [41,42]. Recent work by Andrade-Vieira and colleagues [6] also investigated the role of LKB1 in ErbB2-mediated tumorigenesis using a similar mouse strain (*stk11*^−/−^; NIC). They observed a ~ 25% decrease in tumor latency, which was not apparent in our model. We observed a ~ 8% reduction in median tumor latency that was not statistically significant (Figure 1A). However, detailed whole-mount analysis of our mice revealed the presence and early onset of hyperplastic lesions in the mammary epithelium when LKB1 was absent (Figure 1C). This observation was similar to the growth advantage displayed by LKB1-knockdown NIC tumor cells *in vivo* at early time points (Figure 1D). Thus, despite differences in tumor latency, both models indicate that LKB1 loss can cooperate with ErbB2 to promote breast tumor initiation. Our data suggest that the prominent phenotypic changes associated with LKB1-deletion in breast tumors are a reprogramming of signal transduction and metabolic pathways to favor increased bioenergetic capacity and cell growth.

Our work and that of other groups [43] suggests that loss of LKB1 cooperates with oncogenes to modulate the initiation and growth properties of tumors; however, our data indicate that the impact of reducing LKB1 expression on breast cancer development is complex. LKB1 deletion in mammary tissue promotes the induction of mammary tumors with low penetrance and long latency [4], suggesting that LKB1 deletion or loss-of-heterozygosity may not be a significant driving event for breast cancer. In contrast, deletion of *STK11* in MYC-driven breast tumor models significantly reduced the latency period for tumor development [5]. In the context of tumors driven by unregulated ErbB2 signaling, complete loss of LKB1 does not affect the latency of tumor formation driven by the ErbB2 oncogene; rather, it increases the total number of pre-neoplastic lesions and overt tumors that form in these animals. Thus, while ErbB2 may drive the establishment of primary tumors, increased cell growth, and deregulated metabolism, the differences observed between the ErbB2 model and that of other groups may reflect differences in the mechanisms by which ErbB2 promotes tumor development relative to other oncogenes. Both PI3K, which is activated by ErbB2, and MYC are strong drivers of metabolism; thus loss of LKB1 may synergize specifically with these oncogenes by enhancing pro-growth metabolic pathways.

One of the striking features observed in primary LKB1-deficient ErbB2-postive breast tumors is the amplification of signal transduction pathways impacting cell growth and metabolism. ErbB2 is an oncogenic receptor tyrosine kinase that initiates signaling pathways that control both cell proliferation and survival, including MAPK/ERK and PI3K/Akt pathways [17]. Using RPPA analysis to detect changes in signal transduction pathways in primary breast tumors, we observed major shifts in cellular signaling specifically in primary LKB1-deficient ErbB2-positive breast tumors. Consistent with previously established roles for LKB1, deletion of LKB1 led to decreased AMPK signaling and increased mTORC1 signaling in ErbB2-positive breast tumors. However, we also observed evidence of enhanced signaling by other kinases including Src, MEK1, and MAPK, suggesting a previously unappreciated negative regulatory role of LKB1 on these pathways. Importantly, reducing AMPK activity may not be the only means by which mTOR activity is elevated in LKB1-deficient tumors. Akt is a direct activator of mTOR, and both Akt phosphorylation (T308/S473) and phosphorylation of Akt targets (GSK3β, PRAS40) were elevated in ErbB2-positive tumors lacking LKB1. By removing an endogenous repressor of both mTOR and Akt activity, LKB1 loss may be one way for oncogenic ErbB2 to reprogram signal transduction in tumors to promote metabolism and increased cell growth during transformation.

One of the mechanisms by which oncogenes promote tumor cell growth and proliferation is through enhanced activation of key metabolic pathways, such as glycolysis [44]. Here we show that loss of LKB1 in ErbB2-mediated breast cancer is sufficient to promote the Warburg effect. ErbB2-positive breast cancer cells lacking LKB1 displayed increased expression of several enzymes and transporters that support glycolysis, and both glycolytic flux and overall lactate production were enhanced in LKB1-deficient breast cancer cells. The enhanced glycolytic metabolism observed in LKB1-deficient breast cancer cells was reversed by mTORC1 inhibition, suggesting that elevated mTOR signaling downstream of LKB1 drives the metabolic phenotype of these cancers. We also observed hallmarks of the Warburg effect, notably increased intratumor glucose and lactate levels, in primary LKB1-deficient ErbB2-postive tumors, suggesting that LKB1 regulates glucose metabolism in tumors *in situ*. This is consistent with previous work showing enhanced glucose uptake by fluorodeoxyglucose (^18^F) positron emission tomography in benign *LKB1*^+/−^ colon polyps [45]. Metabolic analysis also revealed that LKB1 loss promotes an increased bioenergetic state in ErbB2-positive tumors; the level of energy storage metabolites, particularly ATP and creatine, were elevated in LKB1-null tumors. Thus, silencing LKB1 may prime breast cancer cells for growth by modulating pro-growth glycolytic metabolism and enhancing ATP production and/or storage.

Given the role of LKB1 as a regulator of several protein kinase pathways, its loss likely affects multiple biological pathways in tumors in addition to metabolism. Epithelial integrity is an important parameter for tissue homeostasis, and loss of epithelial integrity as well as disruption of normal cellular polarization is often a precursor to metastasis [5,29]. Our data suggest that loss of LKB1 leads to altered cell junction formation, reduced expression of epithelial markers, and increased migratory and invasive properties of breast cancer cells *in vitro*. It is unclear whether the metabolic changes induced by LKB1 loss contribute significantly to these phenotypes.

Clinical data shows LKB1 loss in more invasive cancers and *in vitro* data suggests association between loss of LKB1 and acquisition of pro-migratory and pro-invasive properties [46-48], However, despite these pro-growth and pro-metastatic phenotypes, we consistently observed a decrease in the ability of LKB1-deficient breast cancer cells to grow as metastases in the lung (Figure 3), suggesting that LKB1 is required for efficient tumor cell growth in a metastatic setting. The diminished lung metastatic burden in animals with LKB1-null ErbB2 breast tumors raises interesting questions regarding the fitness of LKB1-deficient tumor cells. The metastatic process represents a major energetic stress for the cells as they leave their native environment, travel through the blood, and ultimately seed in a new organ, where they must adapt to a new environment. Recent evidence suggests that LKB1 may be required for primary tumors to adapt to and survive metabolic stress. Models of mutant K-ras-driven lung tumorigenesis demonstrate that LKB1 loss accelerates lung tumor formation [43], but these tumors display increased sensitivity to apoptosis induced by the metabolic stressor phenformin [49]. Consistent with these observations, we find that LKB1-deficient breast cancer cells are increasingly sensitive to glucose limitation. Likewise, LKB1-null NIC cells are unable to adapt their metabolism when challenged with the mitochondrial inhibitor metformin. The mechanisms that underlie this increased sensitivity to metabolic stress are still being examined.

One interesting aspect of the data presented here is that LKB1-deficient breast tumors display heightened energetics at the primary site, but appear to require LKB1 for efficient metastasis to the lung. It is possible that loss of LKB1 locks these cells into a specific mode of pro-growth metabolism, making them less able to adapt to changing tumor or metastatic microenvironments with fluctuating nutrient supply. ErbB2-positive breast tumor cells lacking LKB1 appear to exist in a pro-growth state of growth (that is, elevated Akt/mTOR, increased glycolysis); as demonstrated in Figure 7, these cells continue to maintain a pro-growth state even in the face of reduced nutrient availability. This may explain why dampening the pro-growth state of LKB1-null breast tumor cells, with rapamycin or similar agents, enhances their survival under low glucose conditions. Thus, despite its anti-proliferative effects, LKB1 may confer metabolic flexibility to tumor cells as they colonize and attempt to re-initiate growth in a foreign microenvironment. Collectively our data suggest that in breast cancer LKB1 represents a molecular switch that can regulate breast tumor growth in a stage-dependent manner; loss of LKB1 promotes oncogene-dependent tumorigenesis and early-stage growth in the primary site, but attenuates the growth of breast cancer cells as lung metastases.

## Conclusions

While loss of LKB1 expression has previously been linked to breast cancer, the exact role of LKB1 in regulating breast cancer development and metabolism has remained unclear. Here we demonstrate that loss of LKB1 increases ErbB2-driven mammary tumor initiation and early-stage tumor growth. Reducing LKB1 expression in ErbB2-expressing tumors promotes a pro-growth molecular and phenotypic signature characterized by elevation of Akt and mTORC1 signaling, a metabolic shift towards aerobic glycolysis (Warburg effect), disruption in junctional integrity, and increased migratory and invasive properties *in vitro*. However, despite the pro-growth signature displayed by LKB1-deficient mammary tumors, LKB1-deficient breast tumor cells failed to metastasize the lungs efficiently. We postulate that LKB1 functions as a metabolic master switch in breast cancer. Loss or silencing of LKB1 promotes a switch to the Warburg effect and pro-growth metabolic program to support increased bioenergetic and biosynthetic demand during ErbB2-mediated breast tumor initiation and progression.

## Availability of supporting data

The data sets supporting the results of this article are included within the article (and its additional files).

## Abbreviations

2-NBDG: 2-(N-(7-nitrobenz-2-oxa-1,3-diazol-4-yl)amino)-2-deoxyglucose; 4E-BP1: 4E-binding protein 1; ACC: Acetyl-CoA carboxylase; ACN: Acetonitrile; Akt: Protein kinase B (PKB); AMPK: AMP-activated protein kinase; BRCA1/2: Breast cancer type 1/2 susceptibility protein; DAPI: 4',6-diamidino-2-phenylindole; DMEM: Dulbecco’s modified eagle’s medium; ECAR: Extracellular acidification rate; ErbB2: Human epidermal growth factor receptor 2; ERK: Extracellular-signal-regulated kinases; FBS: Fetal bovine serum; FF: Firefly luciferase; GFP: Green fluorescent protein; GC-MS: Gas chromatography and mass spectrometry; GSK3: Glycogen synthase kinase 3; Hk2: Hexokinase 2; KD: Knockdown; LC-MS: Liquid chromatography and mass spectrometry; LDHA: Lactate dehydrogenase A; LKB1: Liver kinase B1; MAPK: Mitogen-activated protein kinases; MMTV: Mouse mammary tumor virus; mTOR: Mammalian target of rapamycin; mTORC1: mTOR complex 1; OCR: Oxygen consumption rate; PBS: Phosphate-bufferedsaline; PCR: Polymerase chain reaction; PDHK1: Pyruvate dehydrogenase kinase 1; PJS: Peutz-Jeghers syndrome; PI3K: Phosphatidylinositide 3-kinases; qPCR: quantitative PCR; RPPA: Reverse phase protein array; SCID: Severe combined immunodeficiency; shRNA: small hairpin RNA; Src: Sarcoma (proto-oncogene tyrosine-protein kinase); STK11: Serine threonine kinase 11; TSC: Tuberous sclerosis complex.

## Competing interests

The authors declare no conflicts or competing interests.

## Authors’ contributions

The majority of experiments were designed by FD, PMS, and RGJ and executed by FD. The NIC mouse model was developed by WJM and experimental animals generated by FD. Metabolomics experiments were conducted by DA and FD. Respirometry experiments, caspase 3 activity assay, and extracellular lactate measurements were performed by TG and FD. Glucose uptake and 7-AAD staining were performed by JB and FD. Three-dimensional cell culture was performed by CL. Histology and immunohistochemistry were performed by ZD. Tail vein injections were performed by MGA, and RPPA experiments by DRS and GBM. The manuscript was written by FD and edited by FD, PMS, and RGJ. All authors read and approved the final manuscript.

## Supplementary Material

Additional file 1 Supplementary materials and methods, supplemental references, and supplementary figure legends. Click here for file

Additional file 2: Table S1 List of antibodies. Click here for file

Additional file 3: Table S2 List of qPCR primers. Click here for file

Additional file 4: Figure S1 Immunoblot analysis on parental NIC mammary tumor cells(pNIC), NIC tumor cells harboring shRNAs targeting firefly luciferase (NIC-FF) and NIC mammary tumors with stable LKB1 knockdown (NIC-LKB1 KD). NIC mammary tumor explants were serum starved overnight and then stimulated with serum alone or serum combined with metformin (5 mM) for 1 or 6 hours. Immunoblot analysis was performed using antibodies against phospho-AMPK (p-AMPK), total AMPK (AMPK) and phospho-ACC (p-ACC). Immunoblotting for α-tubulin served as a loading control. Click here for file

Additional file 5: Table S3 RPPA data corresponding to the heatmap (126 antibodies). Click here for file

Additional file 6: Figure S2 LKB1 loss confers a pro-growth signal transduction signature in ErbB2-positive mammary tumors. Five NIC/LKB1^+/+^ and five NIC/LKB1^fl/fl ^mammary tumors were subjected to RPPA analysis. Unsupervised hierarchical clustering identifies distinct protein and phospho-protein expression patterns in NIC/LKB1^+/+ ^versus NIC/LKB1^fl/fl^ mammary tumors. The color key indicates level of expression, with green signifying proteins and phospho-proteins that are underexpressed and red identifying those that are overexpressed. Click here for file

Additional file 7: Figure S3 Immunohistochemical staining of mammary tumors arising in NIC/LKB1^+/+^ and NIC/LKB1^fl/fl^ mice, using antibodies against phospho-AMPK (p-AMPK) and total AMPK (AMPK). The scale bar within the upper left inset represents 20 μm and applies to insets in all panels. The scale bar in the upper left panel represents 150 μm and applies to all panels. Click here for file

Additional file 8: Figure S4 Immunohistochemical staining of mammary tumors arising in NIC/LKB1^+/+^ and NIC/LKB1^fl/fl^ mice, using antibodies against phospho-S6 (p-S6), total S6 (S6), phospho-4E-BP1 (p-4E-BP1) and total 4E-BP1 (4E-BP1). The scale bar within the upper left inset represents 20 μm and applies to insets in all panels. The scale bar in the upper left panel represents 150 μm and applies to all panels. Click here for file

Additional file 9: Figure S5 NIC-FF and NIC-LKB1 KD cells were treated with or without rapamycin (100 nM) for 48 hours in 25 mM of glucose, and extracellular levels of lactate were measured in conditioned media using an enzymatic assay (Eton Bioscience kit). The data correspond to one representative experiment from three independent replicate, each performed in triplicate. Click here for file

Additional file 10: Figure S6 Protein extracts were prepared from NIC-FF and NIC-LKB1 KD cells treated with metformin (100 nM) for 6, 12, and 24 hours. Immunoblotting was performed to assess the inhibition of mTOR activity (pS6/S6; mobility shift in 4E-BP1) and the activation of AMPK (pAMK/AMPK) following metformin treatment. Immunoblotting for α-tubulin served as a loading control. Click here for file
