# Supplementary material for: LKB1 is a central regulator of tumor initiation and pro-growth metabolism in ErbB2-mediated breast cancer
Source: Cancer Metab. 2013 Aug 14;1:18. doi: 10.1186/2049-3002-1-18 (PMC4178213; doi:10.1186/2049-3002-1-18)
Supplement: Additional file 1 — Supplementary materials and methods, supplemental references, and supplementary figure legends. [file 2049-3002-1-18-S1.pdf]

Supplementary materials and methods

Immunohistochemical Staining

Tissue fixation and immunohistochemical (IHC) staining was carried out as described (1). The following primary antibodies have been used: Total AMPK (1:100), phospho-AMPK (1:100), total S6 (1:100), phospho-S6 (1:2,000), total 4E-BP1 (1:50) and phospho-4E-BP1 (1:50). Primary antibodies were used in conjunction with the appropriate Biotin-SP-conjugated anti-IgG secondary antibodies (Jackson Laboratories). Tissue sections were developed with 3-3-diaminobenzidine-tetrahydrochloride and counterstained with hematoxylin before being scanned using a Scanscope XT digital slide scanner (Aperio).

Statistical Analysis of the RPPA Data

We performed a two-step QC test. In the first step, QC Scores were determined and compared with visual evaluation of the slides. In the second step, antibody correlation was determined for slides with QC Scores < 0.8 but considered good by visual evaluation; slides with antibody correlation > 0.6 were included for analyses. For antibodies with replicated slides, we chose one with the highest QC Scores. The QC Scores and antibody correlations are included in the data set for your reference. Data for the heatmaps contain the 151 individual antibodies with QC Scores > 0.8 or antibody correlations > 6.0. There were 25 individual mouse antibodies (labeled with "-M") that were removed before creating heatmaps for mouse xenograft samples. Only 126 antibodies were used on heatmaps for mouse xenograft samples.

Supplemental References

1. Tabariès S, Dong Z, Annis MG, Omeroglu A, Pepin F, Ouellet V, et al. Claudin-2 is selectively enriched in and promotes the formation of breast cancer liver metastases through engagement of integrin complexes. Oncogene. 2011;30(11):1318-28. Epub 2010/11/16.

Supplementary Figure Legends

Additional file 1: Figure S1: Immunoblot analysis on parental NIC mammary tumor cells (pNIC), NIC tumor cells harboring shRNAs targeting FireFly luciferase (NIC-FF) and NIC mammary tumors with stable LKB1 knock-down (NIC-LKB1 KD). NIC mammary tumor explants were serum starved overnight and then stimulated with serum alone or serum combined with metformin (5mM) for 1 or 6 hours. Immunoblot analysis was performed using antibodies against phospho-AMPK (p-AMPK), total AMPK (AMPK) and phospho-ACC (p-ACC). Immunoblotting for α-Tubulin served as a loading control.

Additional file 2: Figure S2: LKB1 loss confers a pro-growth signal transduction signature in ErbB2-positive mammary tumors. Five NIC/LKB1$^{+/+}$ and five NIC/LKB1$^{fl/fl}$ mammary tumors were subjected to Reverse Phase Protein Array (RPPA) analysis. Unsupervised hierarchical clustering identifies distinct protein and phospho-protein expression patterns in NIC/LKB1$^{+/+}$ versus NIC/LKB1$^{fl/fl}$ mammary tumors. The color key indicates level of expression, with green signifying proteins and phospho-proteins that are underexpressed and red identifying those that are overexpressed.

Additional file 3: Figure S3: Immunohistochemical staining of mammary tumors arising in NIC/LKB1$^{+/+}$ and NIC/LKB1$^{fl/fl}$ mice, using antibodies against phospho-AMPK (p-AMPK) and total AMPK (AMPK). The scale bar within the upper left inset represents 20µm and applies to all panels. The scale bar in the upper left panel represents 150µm and applies to all panels.

Additional file 4: Figure S4: Immunohistochemical staining of mammary tumors arising in NIC/LKB1$^{+/+}$ and NIC/LKB1$^{fl/fl}$ mice, using antibodies against phospho-S6 (p-S6), total S6 (S6), phosph-4E-BP1 (p-4E-BP1) and total 4E-BP1 (4E-BP1). The scale bar within the upper left inset represents 20µm and applies to all panels. The scale bar in the upper left panel represents 150µm and applies to all panels.

Additional file 5: Figure S5: NIC-FF and NIC-LKB1 KD cells were treated with or without Rapamycin (100nM) for 48 hours in 25mM of glucose, and extracellular levels of lactate were measured in conditioned media using an enzymatic assay (Eton Bioscience kit). The data correspond to one representative experiment from three independent replicate, each performed in triplicate.

Additional file 6: Figure S6: Protein extracts were prepared from NIC-FF and NIC-LKB1 KD cells treated with Metformin (100nM) for 6, 12 and 24 hours. Immunoblotting was performed assess the inhibition of mTOR activity (pS6/S6; mobility shift in 4E-BP1) and the activation of AMPK (pAMK/AMPK) following Metformin treatment. Immunoblotting for α-Tubulin served as a loading control.
