# Supplementary material for: LKB1 is a central regulator of tumor initiation and pro-growth metabolism in ErbB2-mediated breast cancer
Source: Cancer Metab. 2013 Aug 14;1:18. doi: 10.1186/2049-3002-1-18 (PMC4178213; doi:10.1186/2049-3002-1-18)
Supplement: Additional file 2: Table S1 — List of antibodies. [file 2049-3002-1-18-S2.pptx]

## Slide 1
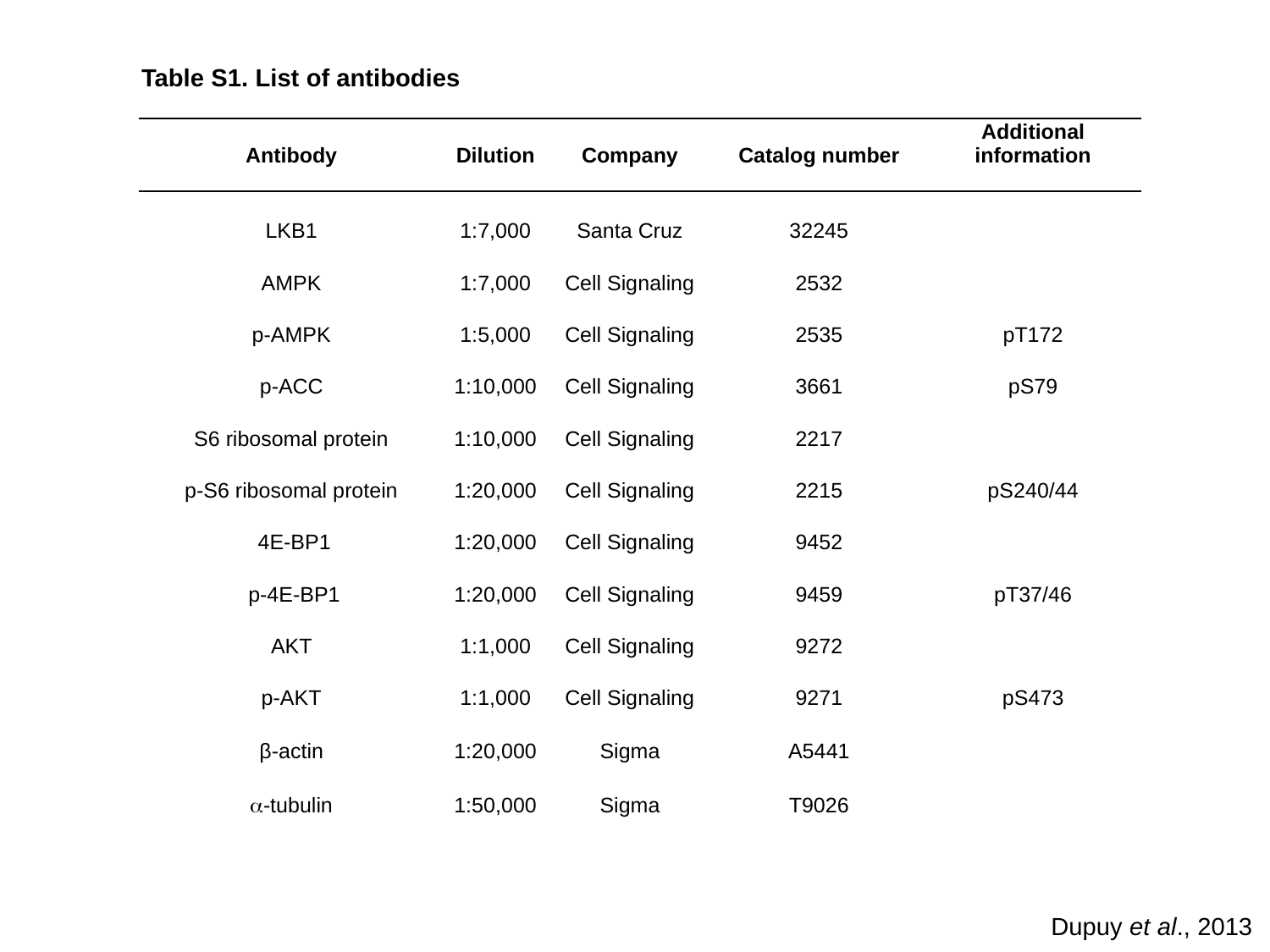

Table S1. List of antibodies
| Antibody | Dilution | Company | Catalog number | Additional information |
| --- | --- | --- | --- | --- |
| | | | | |
| LKB1 | 1:7,000 | Santa Cruz | 32245 | |
| | | | | |
| AMPK | 1:7,000 | Cell Signaling | 2532 | |
| | | | | |
| p-AMPK | 1:5,000 | Cell Signaling | 2535 | pT172 |
| | | | | |
| p-ACC | 1:10,000 | Cell Signaling | 3661 | pS79 |
| | | | | |
| S6 ribosomal protein | 1:10,000 | Cell Signaling | 2217 | |
| | | | | |
| p-S6 ribosomal protein | 1:20,000 | Cell Signaling | 2215 | pS240/44 |
| | | | | |
| 4E-BP1 | 1:20,000 | Cell Signaling | 9452 | |
| | | | | |
| p-4E-BP1 | 1:20,000 | Cell Signaling | 9459 | pT37/46 |
| | | | | |
| AKT | 1:1,000 | Cell Signaling | 9272 | |
| | | | | |
| p-AKT | 1:1,000 | Cell Signaling | 9271 | pS473 |
| | | | | |
| β-actin | 1:20,000 | Sigma | A5441 | |
| | | | | |
| α-tubulin | 1:50,000 | Sigma | T9026 | |
| | | | | |
Dupuy et al., 2013
