# Supplementary material for: LKB1 is a central regulator of tumor initiation and pro-growth metabolism in ErbB2-mediated breast cancer
Source: Cancer Metab. 2013 Aug 14;1:18. doi: 10.1186/2049-3002-1-18 (PMC4178213; doi:10.1186/2049-3002-1-18)
Supplement: Additional file 3: Table S2 — List of qPCR primers. [file 2049-3002-1-18-S3.pptx]

## Slide 1
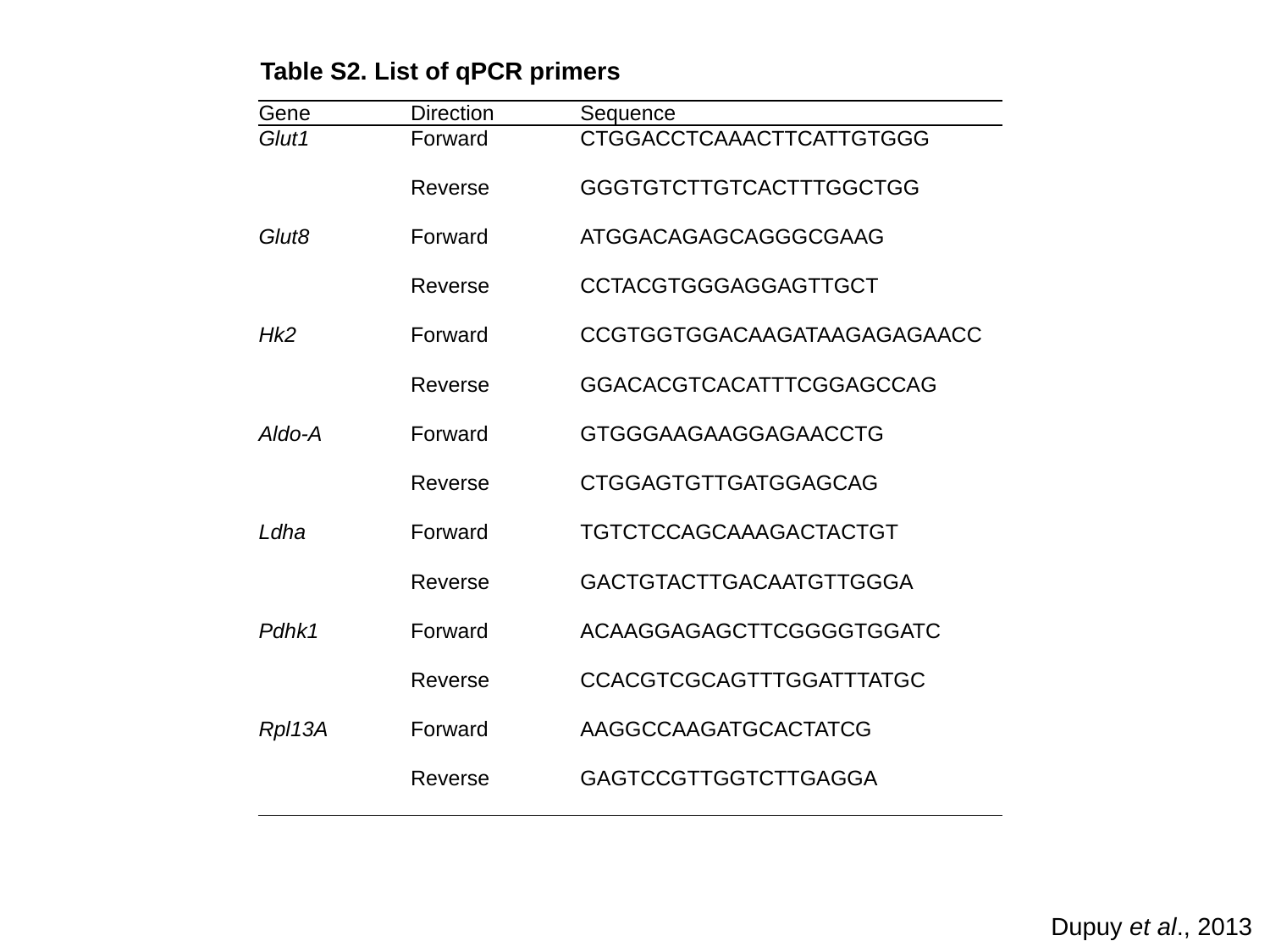

Table S2. List of qPCR primers
| | | |
| --- | --- | --- |
| Gene | Direction | Sequence |
| Glut1 | Forward | CTGGACCTCAAACTTCATTGTGGG |
| | | |
| | Reverse | GGGTGTCTTGTCACTTTGGCTGG |
| | | |
| Glut8 | Forward | ATGGACAGAGCAGGGCGAAG |
| | | |
| | Reverse | CCTACGTGGGAGGAGTTGCT |
| | | |
| Hk2 | Forward | CCGTGGTGGACAAGATAAGAGAGAACC |
| | | |
| | Reverse | GGACACGTCACATTTCGGAGCCAG |
| | | |
| Aldo-A | Forward | GTGGGAAGAAGGAGAACCTG |
| | | |
| | Reverse | CTGGAGTGTTGATGGAGCAG |
| | | |
| Ldha | Forward | TGTCTCCAGCAAAGACTACTGT |
| | | |
| | Reverse | GACTGTACTTGACAATGTTGGGA |
| | | |
| Pdhk1 | Forward | ACAAGGAGAGCTTCGGGGTGGATC |
| | | |
| | Reverse | CCACGTCGCAGTTTGGATTTATGC |
| | | |
| Rpl13A | Forward | AAGGCCAAGATGCACTATCG |
| | | |
| | Reverse | GAGTCCGTTGGTCTTGAGGA |
| | | |
Dupuy et al., 2013
