# Supplementary material for: LKB1 is a central regulator of tumor initiation and pro-growth metabolism in ErbB2-mediated breast cancer
Source: Cancer Metab. 2013 Aug 14;1:18. doi: 10.1186/2049-3002-1-18 (PMC4178213; doi:10.1186/2049-3002-1-18)
Supplement: Additional file 4: Figure S1 — Immunoblot analysis on parental NIC mammary tumor cells(pNIC), NIC tumor cells harboring shRNAs targeting firefly luciferase (NIC-FF) and NIC mammary tumors with stable LKB1 knockdown (NIC-LKB1 KD). NIC mammary tumor explants were serum starved overnight and then stimulated with serum alone or serum combined with metformin (5 mM) for 1 or 6 hours. Immunoblot analysis was performed using antibodies against phospho-AMPK (p-AMPK), total AMPK (AMPK) and phospho-ACC (p-ACC). Immunoblotting for α-tubulin served as a loading control. [file 2049-3002-1-18-S4.pptx]

## Slide 1
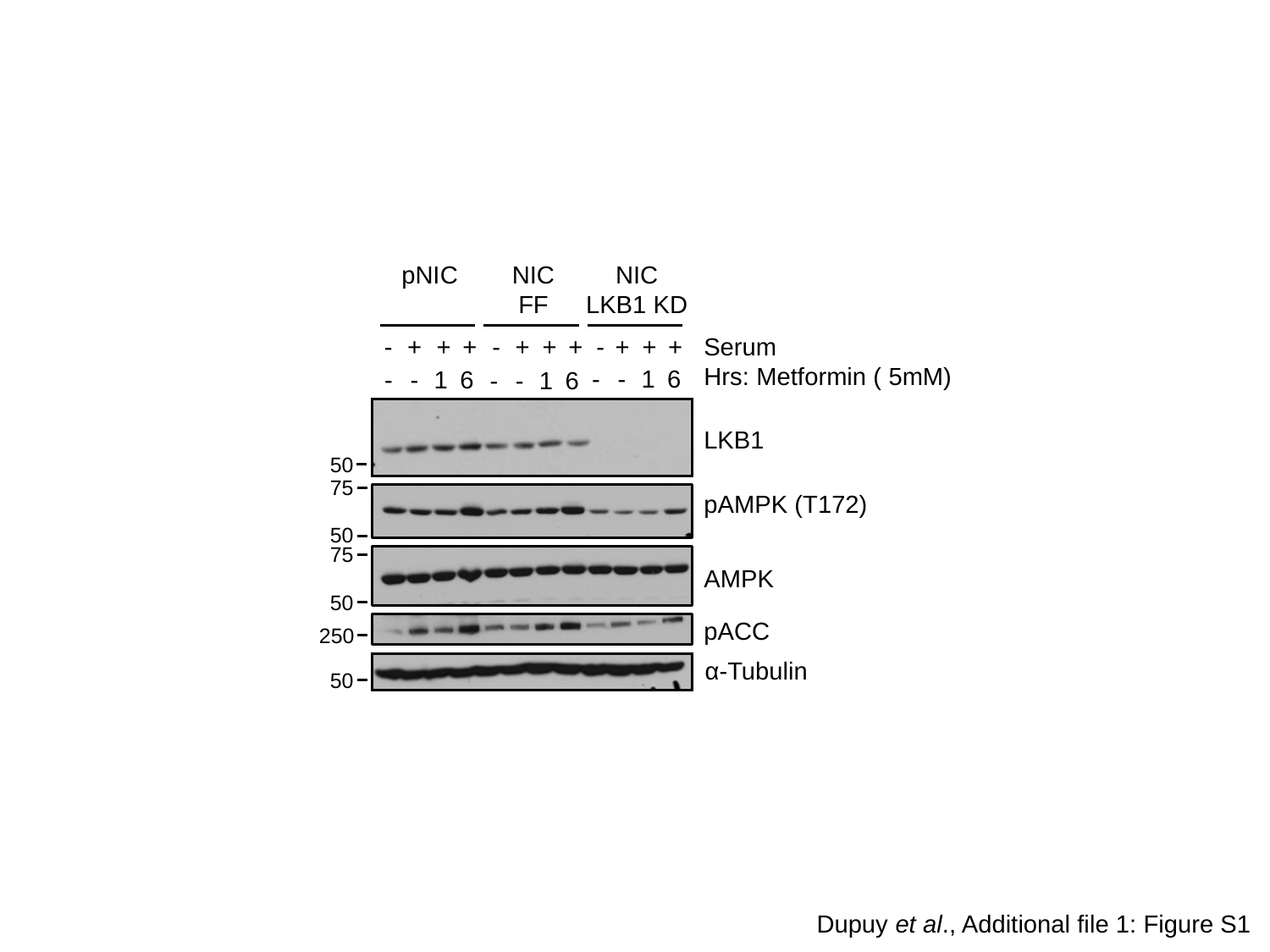

pNIC
NIC
FF
NIC
LKB1 KD
-
+
+
+
-
+
+
+
-
+
+
+
Serum
Hrs: Metformin ( 5mM)
-
-
1
6
-
-
1
6
-
-
1
6
LKB1
50
75
pAMPK (T172)
50
75
AMPK
50
pACC
250
α-Tubulin
50
Dupuy et al., Additional file 1: Figure S1
