# Supplementary figures and images for: LKB1 is a central regulator of tumor initiation and pro-growth metabolism in ErbB2-mediated breast cancer
Source: Cancer Metab. 2013 Aug 14;1:18. doi: 10.1186/2049-3002-1-18 (PMC4178213; doi:10.1186/2049-3002-1-18)

## Slide 1
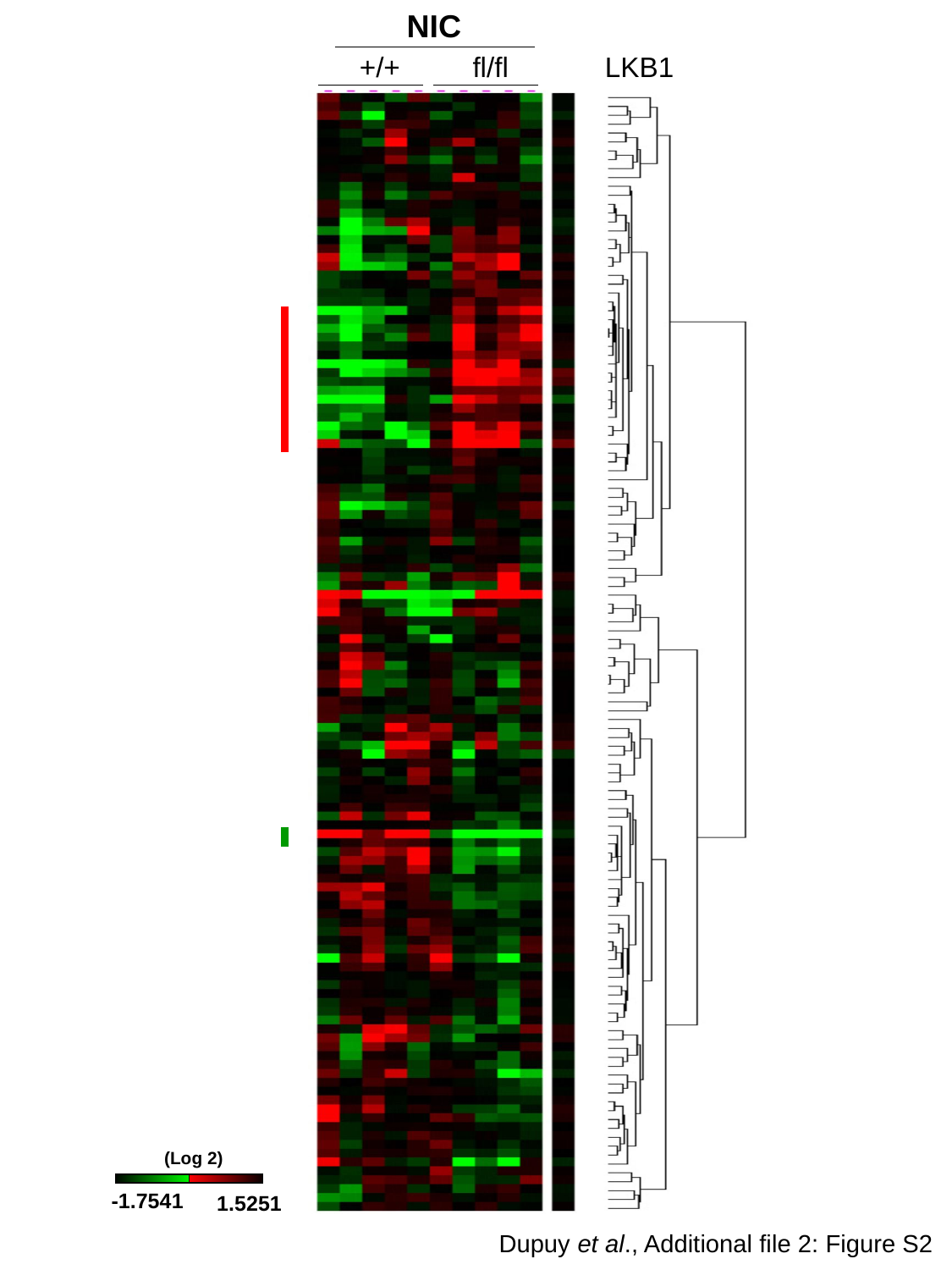

NIC
+/+
fl/fl
LKB1
1.5251
(Log 2)
-1.7541
Dupuy et al., Additional file 2: Figure S2

Supplement: Additional file 6: Figure S2 — LKB1 loss confers a pro-growth signal transduction signature in ErbB2-positive mammary tumors. Five NIC/LKB1+/+ and five NIC/LKB1fl/fl mammary tumors were subjected to RPPA analysis. Unsupervised hierarchical clustering identifies distinct protein and phospho-protein expression patterns in NIC/LKB1+/+ versus NIC/LKB1fl/fl mammary tumors. The color key indicates level of expression, with green signifying proteins and phospho-proteins that are underexpressed and red identifying those that are overexpressed. [file 2049-3002-1-18-S6.pptx]

## Slide 1
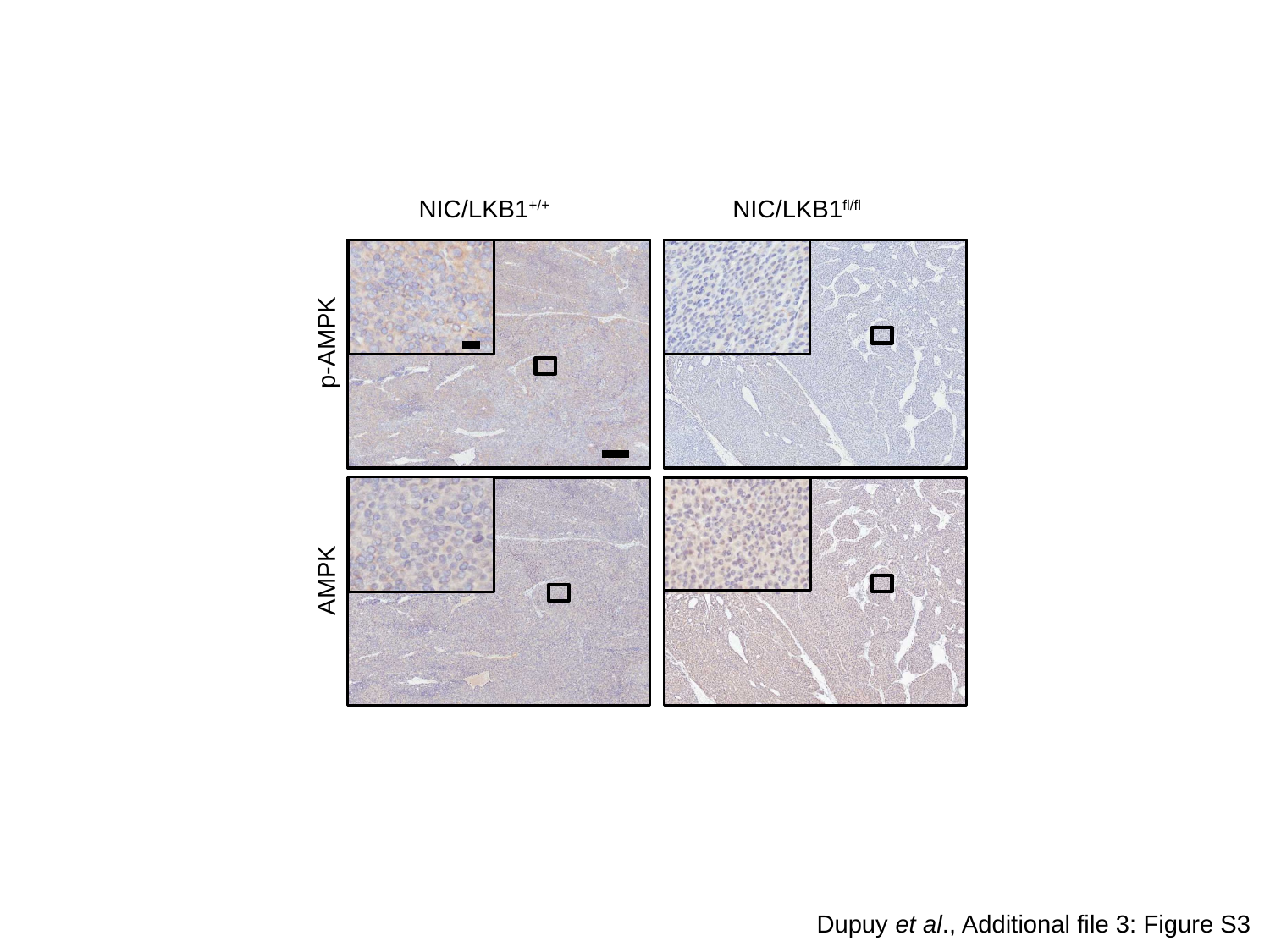

NIC/LKB1+/+
NIC/LKB1fl/fl
p-AMPK
AMPK
Dupuy et al., Additional file 3: Figure S3

Supplement: Additional file 7: Figure S3 — Immunohistochemical staining of mammary tumors arising in NIC/LKB1+/+ and NIC/LKB1fl/fl mice, using antibodies against phospho-AMPK (p-AMPK) and total AMPK (AMPK). The scale bar within the upper left inset represents 20 μm and applies to insets in all panels. The scale bar in the upper left panel represents 150 μm and applies to all panels. [file 2049-3002-1-18-S7.pptx]

## Slide 1
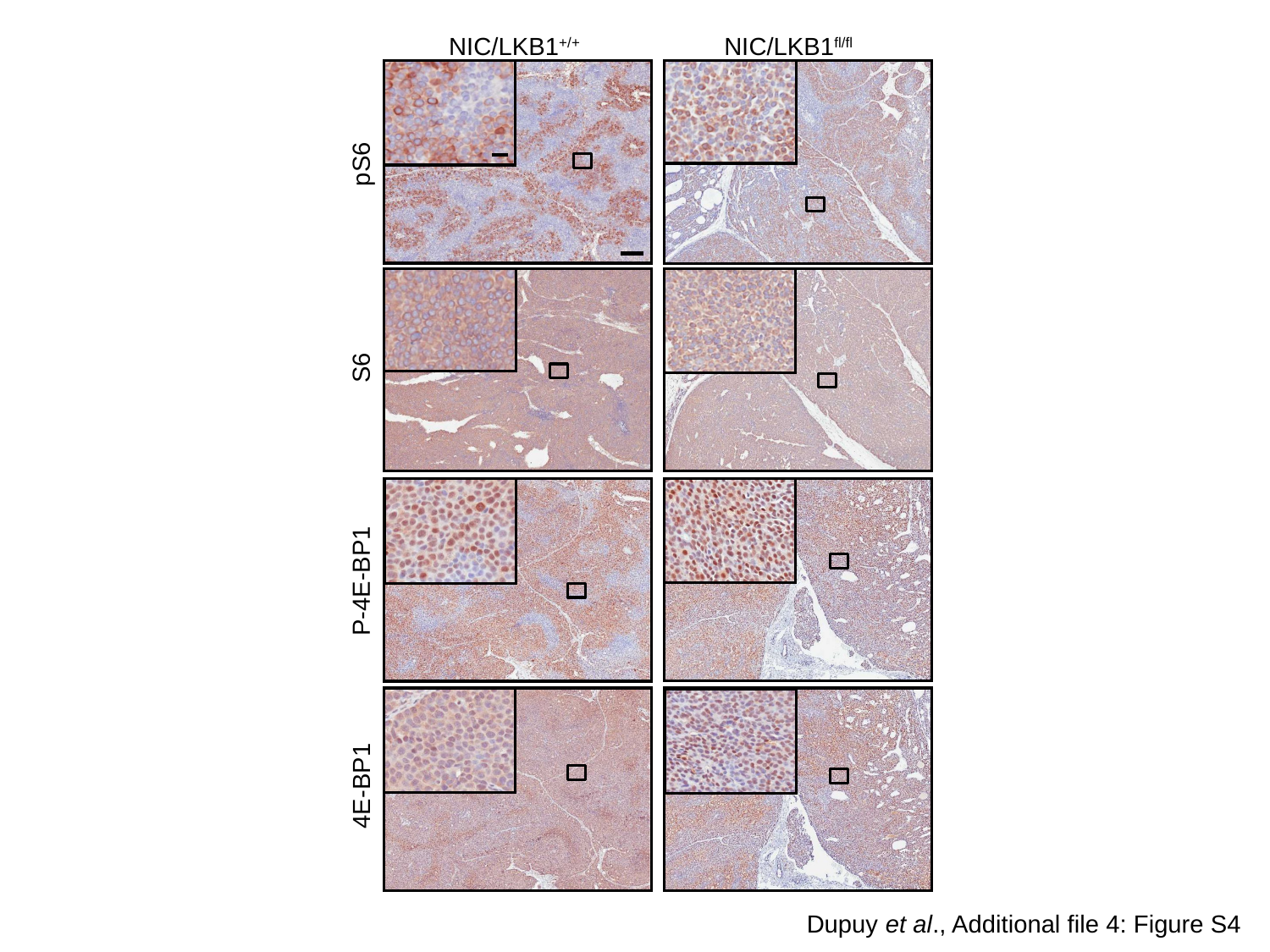

NIC/LKB1+/+
NIC/LKB1fl/fl
pS6
S6
P-4E-BP1
4E-BP1
Dupuy et al., Additional file 4: Figure S4

Supplement: Additional file 8: Figure S4 — Immunohistochemical staining of mammary tumors arising in NIC/LKB1+/+ and NIC/LKB1fl/fl mice, using antibodies against phospho-S6 (p-S6), total S6 (S6), phospho-4E-BP1 (p-4E-BP1) and total 4E-BP1 (4E-BP1). The scale bar within the upper left inset represents 20 μm and applies to insets in all panels. The scale bar in the upper left panel represents 150 μm and applies to all panels. [file 2049-3002-1-18-S8.pptx]
