# Supplementary material for: LKB1 is a central regulator of tumor initiation and pro-growth metabolism in ErbB2-mediated breast cancer
Source: Cancer Metab. 2013 Aug 14;1:18. doi: 10.1186/2049-3002-1-18 (PMC4178213; doi:10.1186/2049-3002-1-18)
Supplement: Additional file 9: Figure S5 — NIC-FF and NIC-LKB1 KD cells were treated with or without rapamycin (100 nM) for 48 hours in 25 mM of glucose, and extracellular levels of lactate were measured in conditioned media using an enzymatic assay (Eton Bioscience kit). The data correspond to one representative experiment from three independent replicate, each performed in triplicate. [file 2049-3002-1-18-S9.pptx]

## Slide 1
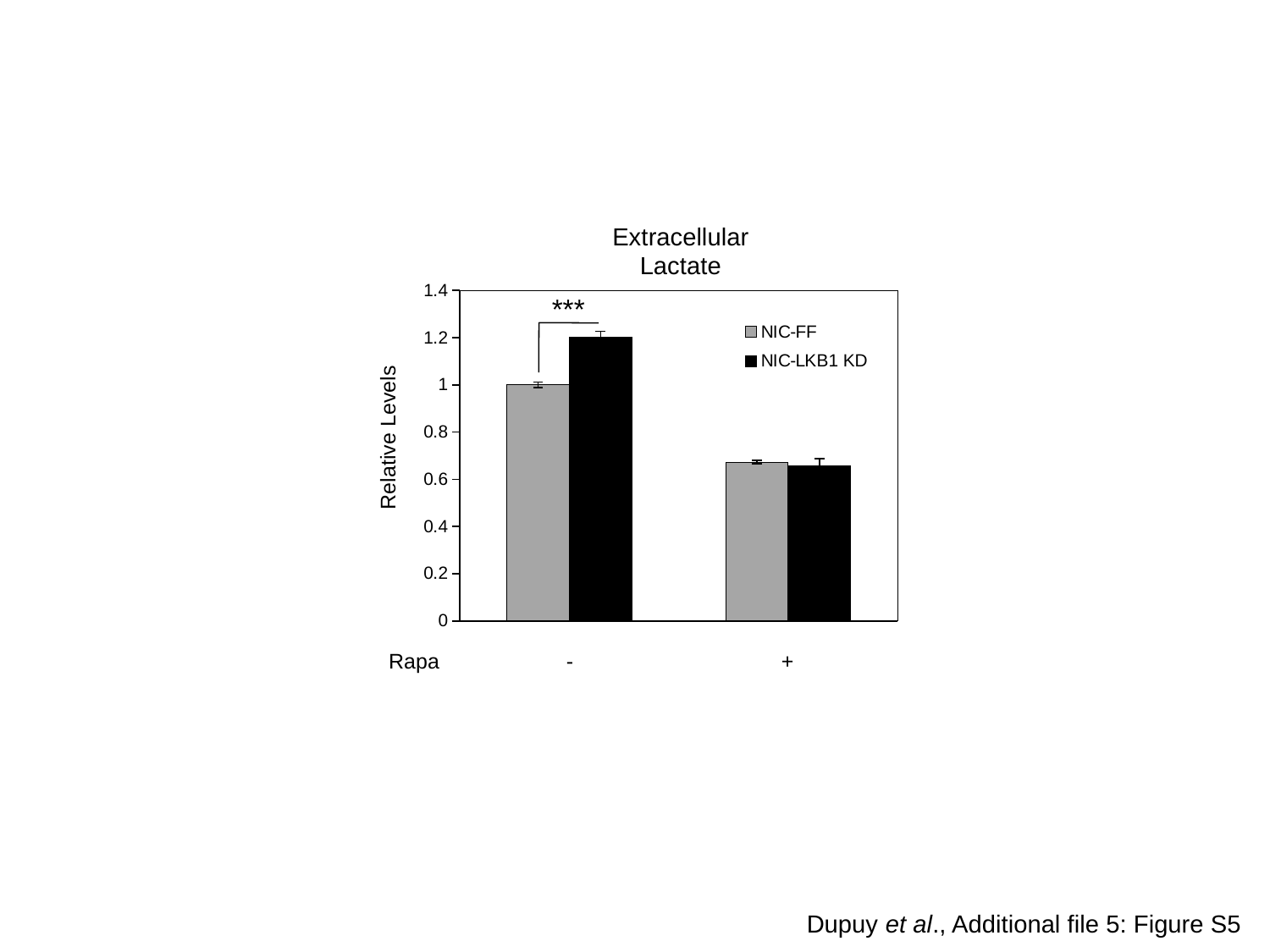

Extracellular Lactate
### Chart
| Category | NIC-FF | NIC-LKB1 KD |
|---|---|---|
| no treatment | 1.0 | 1.202429421127587 |
| rapamycin | 0.672795955290477 | 0.656851388805777 |***
Relative Levels
Rapa
-
+
Dupuy et al., Additional file 5: Figure S5
