# Supplementary material for: LKB1 is a central regulator of tumor initiation and pro-growth metabolism in ErbB2-mediated breast cancer
Source: Cancer Metab. 2013 Aug 14;1:18. doi: 10.1186/2049-3002-1-18 (PMC4178213; doi:10.1186/2049-3002-1-18)
Supplement: Additional file 10: Figure S6 — Protein extracts were prepared from NIC-FF and NIC-LKB1 KD cells treated with metformin (100 nM) for 6, 12, and 24 hours. Immunoblotting was performed to assess the inhibition of mTOR activity (pS6/S6; mobility shift in 4E-BP1) and the activation of AMPK (pAMK/AMPK) following metformin treatment. Immunoblotting for α-tubulin served as a loading control. [file 2049-3002-1-18-S10.pptx]

## Slide 1
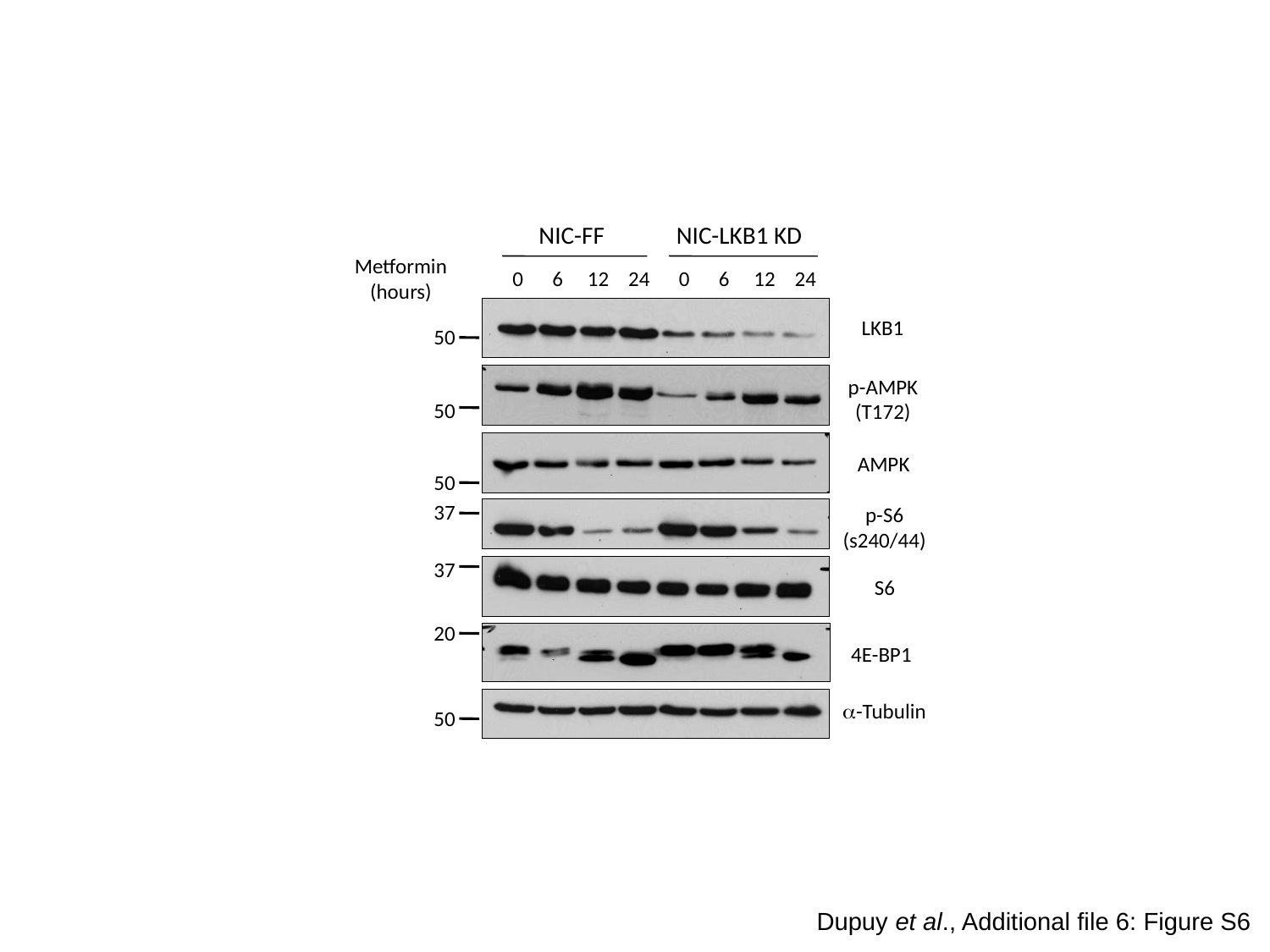

NIC-FF
NIC-LKB1 KD
Metformin (hours)
 0 6 12 24 0 6 12 24
LKB1
50
p-AMPK
(T172)
50
AMPK
50
37
p-S6
(s240/44)
37
S6
20
4E-BP1
α-Tubulin
50
Dupuy et al., Additional file 6: Figure S6
